# Altered brain rhythms and behaviour in the accelerated ovarian failure mouse model of human menopause

**DOI:** 10.1093/braincomms/fcac166

**Published:** 2022-06-22

**Authors:** Sophia Vrontou, Alexis Bédécarrats, Xiaofei Wei, Morikeoluwa Ayodeji, Attila Brassai, László Molnár, Istvan Mody

**Affiliations:** Department of Neurology, The David Geffen School of Medicine at UCLA, Los Angeles, CA 90095, USA; Department of Neurology, The David Geffen School of Medicine at UCLA, Los Angeles, CA 90095, USA; Department of Neurology, The David Geffen School of Medicine at UCLA, Los Angeles, CA 90095, USA; The Peddie School, Hightstown, NJ 08520, USA; Department of Pharmacology, George Emil Palade University of Medicine, Pharmacy, Sciences and Technology, Târgu Mureş 540139, Romania; Department of Electrical Engineering, Sapientia Hungarian University of Transylvania, Târgu Mureş 540485, Romania; Department of Neurology, The David Geffen School of Medicine at UCLA, Los Angeles, CA 90095, USA; Department of Physiology, The David Geffen School of Medicine at UCLA, Los Angeles, CA 90095, USA

**Keywords:** menopause, 4-vinylcyclohexene-diepoxide (VCD), oscillations, hippocampus, sleep

## Abstract

To date, potential mechanisms of menopause-related memory and cognitive deficits have not been elucidated. Therefore, we studied brain oscillations, their phase–amplitude coupling, sleep and vigilance state patterns, running wheel use and other behavioural measures in a translationally valid mouse model of menopause, the 4-vinylcyclohexene-diepoxide-induced accelerated ovarian failure. After accelerated ovarian failure, female mice show significant alterations in brain rhythms, including changes in the frequencies of θ (5–12 Hz) and γ (30–120 Hz) oscillations, a reversed phase–amplitude coupling, altered coupling of hippocampal sharp-wave ripples to medial prefrontal cortical sleep spindles and reduced δ oscillation (0.5–4 Hz) synchrony between the two regions during non-rapid eye movement sleep. In addition, we report on significant circadian variations in the frequencies of θ and γ oscillations, and massive synchronous δ oscillations during wheel running. Our results reveal novel and specific network alterations and feasible signs for diminished brain connectivity in the accelerated ovarian failure mouse model of menopause. Taken together, our results may have identified changes possibly responsible for some of the memory and cognitive deficits previously described in this model. Corresponding future studies in menopausal women could shed light on fundamental mechanisms underlying the neurological and psychiatric comorbidities present during this important transitional phase in women’s lives.

## Teaser

Vrontou *et al*. report novel and specific brain rhythm alterations and signs of decreased brain regional connectivity in the accelerated ovarian failure mouse model of human menopause. These changes could be responsible for the memory and cognitive deficits previously described in this model, and ultimately, also in menopausal women.

## Graphical Abstract

**Graphical Abstract:**
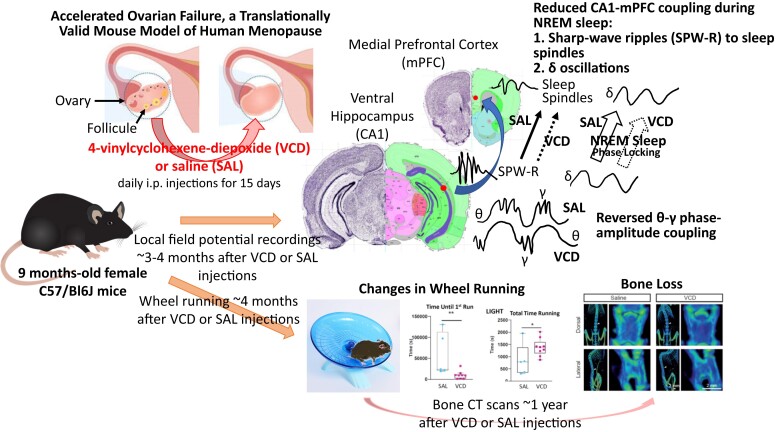

## Introduction

In the context of a global ageing population and the protracted life expectancy of women, it can be expected that the menopausal state will occupy one-third or more of women’s lifetime. Worldwide by 2030, more than 1.2 billion women will be >50 years old. Every single day in the USA, ∼6000 women reach menopause. That is nearly 2.2 million per year, and by the end of 2020, this translates to >45 million women >55 years of age.^1^ Among other findings, this reproductive ageing is associated with decreased levels of ovarian steroids and an elevation of plasma follicle-stimulating hormone (FSH). These hormonal changes take years to stabilize and are most marked during the 2 years prior to, and the 2 years after, the final menstrual period. Many studies have implied that the decline in oestrogens is associated with memory impairments and cognitive decline,^2–5^ particularly those functions involving the prefrontal cortex (PFC), but a meta-analysis of these studies has not been performed until very recently.^6^ The conclusions of this meta-analysis were that distinct stages of the menopause (pre-, peri- and post-menopause) are associated with decreases in working- and delayed verbal memory, and the post-menopausal stage is associated with a decreased phonemic verbal fluency. Additionally, all three stages of menopause are unequivocally associated with a significantly increased risk of developing depression.^6–10^ Moreover, there is a significant body of clinical literature indicating *de novo* sleep disturbances arising during the menopause transition and in the post-menopausal life stage.^10–13^

The majority of women enter menopause via a gradual and irreversible process (peri-menopause) of reduction in ovarian function and decline in oestrogen levels followed by a decrease in oestrogen receptor expression over several years. The duration of peri-menopause is ∼5 years, between ages of 45 and 54, which is followed by amenorrhoea and then post-menopause.^14–16^ Women constitute nearly 70% of the affected Alzheimer’s disease population,^17^ ∼3.5 million Americans aged 65 or older. Alzheimer’s disease prevalence is two to three times higher in post-menopausal women than in men, even after controlling for lifespan.^17,18^ Furthermore, women present with a faster pathology progression and greater memory impairment than men.^17,18^

Memory and cognitive processes are inexorably linked to various brain oscillations.^19^ The γ oscillations are a periodic network activity (30–120 Hz) present in different brain areas during certain wakefulness states and rapid eye movement (REM) sleep and arise from a synchronized excitation and inhibition loop between principal cells and parvalbumin-expressing interneurons (PV + INs), which have a critical role in initiating and maintaining local oscillations.^20–24^ These oscillations are thought to enable encoding and memory formation in discrete neuronal networks, facilitate spike-time-dependent plasticity and are considered to play an important role in the physiology of learning and memory.^25–29^ In addition to γ oscillations, a key network pattern in hippocampus-dependent memory consolidation is the sharp-wave ripple (SPW-R) complex, mostly seen during slow-wave sleep, immobility and consummatory behaviours.^30,31^ The sharp wave (SPW) component of the oscillations reflects the compound depolarization of CA1 neurons triggered by the concurrent activity of multiple reciprocally connected CA3 pyramidal cells (PCs).^30^ This in turn induces a local, fast oscillatory event in the CA1 region, the ‘ripple’ (140-200 Hz),^32,33^ with its frequency depending on the magnitude of the SPW. The cycles of the local field potential (LFP) ripple coincide with the sequential activity of neurons, the identity of which is influenced by previous experience.^31,34^ This neuronal sequence is often similar to place cell sequences observed during exploration.^35–37^ Inhibition of SPW-R after learning results in impairment of memory performance.^38,39^ The SPW-R have a critical role in information transfer between the hippocampus and neocortex and for memory consolidation,^40^ but the local network mechanisms underlying the generation of ripples are not fully understood.^41^ It is clear, however, that just like for γ oscillations, PV + INs play a critical role in the local generation of SPW-R.^42–45^

Although the link between the transition to menopause and diminished cognitive performance is established,^2–5,46,47^ there is a remarkable paucity of studies on possible alterations in brain oscillatory activity known to be involved in cognitive and memory processes. One of the few studies only addressed low-frequency oscillations in a limited number of subjects.^48^ Even more surprising is the lack of such studies in animal models of menopause, most notably in the accelerated ovarian failure (AOF) model that successfully replicates the human peri- and post-menopause stages, including irregular oestrous fluctuations.^49^ This model was developed over 20 years ago,^50–53^ but we still know very little about brain network alterations in rodents that undergo hormonal and metabolic changes equivalent to the human menopause. We have undertaken the present study to address the gap in knowledge about potential alterations in brain oscillations related to memory and cognition in the AOF mouse model of human menopause. Our findings are consistent with significant changes in network activity between ageing mice that have undergone 4-vinylcyclohexene-diepoxide (VCD)-induced ovarian failure compared with age-matched counterparts, and open new perspectives for understanding the alterations in menopause-related changes in cognitive performance, memory and anxiety.

## Materials and methods

### Experimental model and subject details

All procedures were performed in accordance with protocols approved by the UCLA Institutional Animal Care and Use Committee (IACUC) and guidelines of the National Institutes of Health. Mouse strains used were *C57BL/6J* (Black 6, Jackson Laboratory; JAX). Only female mice were used, aged 36 weeks at the time of VCD injections (Supplementary Fig. 1) This late age point of VCD injection was chosen to better recapitulate the menopausal alterations as previous studies injected VCD at much younger ages (8–10 weeks). Mice were group-housed in plastic cages with disposable bedding on a standard 12 h light cycle (6 AM to 6 PM lights on). Animals were randomly allocated into two groups injected with either saline (SAL) alone (*n* = 8) in a SAL group or with 4-vinylcyclohexene diepoxide (VCD; Merck-Sigma-Aldrich) diluted in SAL in a VCD group (*n* = 12). A stock VCD solution of 1094 mg/ml was prepared in sterile 0.9% NaCl (SAL) and was diluted 1/25 for final injection resulting in a VCD concentration of 4.2 mg/0.1 ml in order to achieve a high dose of VCD in each mouse. For the two groups, 0.2 ml of SAL or 0.2 ml of the VCD solution was injected intraperitoneally once a day for 15 days. Based on the average weight of our mice during the entire time of the VCD injections (mean ± SD: 24.16 ± 1.94 g, *n* = 12), the animals received 8.4 mg/mouse/day, the equivalent of a VCD daily dose of 347.7 ± 25.9 mg/kg. During the VCD injections, the mice were housed individually in plastic disposable cages in an isolated Division of Laboratory Animal Medicine (DLAM) facility for injection of toxic chemicals. Our VCD dose is relatively higher than that used in previous studies, most likely due to the advanced age of our animals and the vehicle (SAL), instead of the mineral oil used in previous reports. Of the eight animals in the SAL group, we used six for chronic electrode implants of which two died during the postoperative recovery, leaving four electrode-implanted SAL animals. Of these, all data from one animal were discarded due to highly irregular light/dark (L–D) period activity cycling that was not in line with the nocturnal activity pattern of the rest of the animals, thus leaving three implanted and two unimplanted SAL animals for our studies. Similarly, of the 12 VCD-treated mice, 8 animals were implanted with electrodes. In this group, there were also two attritions during recovery from surgery, and one animal had highly irregular L–D activity cycles and were excluded from the study, leaving five implanted and five unimplanted VCD mice for our studies.

### Electrode fabrication for *in vivo* recordings

Electrodes (Plastics One) were stainless steel, with polyimide electrode insulation, ending in a socket that fitted into the custom-made recording system. Electrode lengths (measured from the bottom of socket contact to the tip of the wire) were 5 mm (for the two unilaterally implanted electrodes) and 25 mm for the ground/reference electrode. Electrode diameters were 125 μm bare and 150 μm insulated. The two unilaterally implanted electrodes were custom-made as follows: a socket (gold-plated stainless steel Amphenol contact, O.D: 1.07 mm, ID: 0.686 mm and length: 7.87 mm) was attached to a flexible 10 mm long PFA Teflon insulated wire. The free tip of this wire was soldered to another 3 mm long Amphenol socket attached to the 5 mm electrode. The openings of the sockets were trimmed to 1 mm length, cleaned from debris, smoothened on the edges and tightened. The three electrodes were assembled into one unit by connecting their sockets with J-B Weld™ two-part epoxy. The space between the sockets corresponded to the distance between the three Amphenol pins soldered to the recording preamplifier.

### Stereotaxic surgery

Female mice 48–49 weeks of age (10–11 weeks after the last VCD injection; Supplementary Fig. 1) were anaesthetized with isoflurane and placed on the stereotaxic apparatus without the ear bars. Retro-orbital sampling was done as described in the Supplementary material. After blood collection, eye ointment was applied to the eyes, the ear bars were put in place and the mouse was positioned in the stereotaxic apparatus. The shaved scalp was disinfected with betadine+70% ethanol and removed along with the periosteum. A solution of 3% H_2_O_2_ in SAL was applied to the exposed skull, which was then cleaned by SAL, dried and gently scored with a scalpel blade. The skull was then covered with a thin layer of cyanoacrylate glue. The two recording electrodes were implanted in the mPFC coordinates (in mm) 1.5 AP, 0.2 ML, 3 DV and ‘vHIP CA1’ at −3.3 AP, 3.5 ML, 3 DV (AP, anterior–posterior; ML, medial–lateral; DV, dorsal–ventral) according to the established coordinates^54^ and from the ALLEN Mouse Brain Atlas, Version 2 (2011), Allen Institute for Brain Science (Supplementary Fig. 2). The vCA1 coordinates consistently positioned the electrodes in the PC layer or slightly above it, as judged by the depth profile of the relationship between θ phase and γ oscillations^55^ in SAL mice and the shape of the recorded SPW-R complexes^40^ (Supplementary Fig. 3B and C) in all SAL and VCD animals (also see Fig. 5A and B). The electrical reference/ground electrode was implanted over the cerebellum. The skull and the lower part of the electrode unit system were then covered with Ortho-Jet™ acrylic resin. During the surgery, lidocaine (2 µl from 2% solution) was injected subcutaneously on the neck and the non-steroidal anti-inflammatory drug Rimadyl (Carprofen) (0.1 mg/kg) was administered intraperitoneally for pain management (Rimadyl was additionally administered for two consecutive days). In addition, 50 µl sterile SAL was administered subcutaneously for hydration during surgery. During the recovery after surgery (1 week), mice were single housed in plastic disposable cages with a plastic lid that did not interfere with the head implants.

### *In vivo* recordings and data acquisition

The electrical signals from the mouse brain were relayed through a custom-made recording system to a computer, located outside the recording room. Briefly, 1–1.5 m long cable was constructed using eight intertwined bare copper wires (single 8058-Magnet Wire) and placed inside a PVC tubing. The cable was soldered accordingly to an LT1112S8#PBF General Purpose Amplifier 2 Circuit 8-SO (Digi-Key) on one end and to an MMA25-011 connector plug with male pins (Digi-Key) to the other end. The connector plug end of the cable was attached to a 10-channel slip ring commutator (Campden Instruments). Both ends of the cable were covered with J-B Weld™ two-part epoxy. The commutator was joined to a custom-made connector box that also contained two 9 V batteries providing the power supply to the preamplifier. The signal was then transmitted to Model 3600 16-channel amplifier (A-M Systems) (LP: 300 Hz, HP: 0.3 Hz, Gain:1K). The output signal was led to an USB 6009 14 bit A/D converter (National Instruments) connected to a laptop computer. Igor 8.0 (Wavemetrics) NIDAQ tools were used to record the two channels in each mouse at a sampling rate of 2048 s^−1^. During the continuous recording sessions (∼21 days) that started at 11–12 weeks after the last VCD injection, the mice were housed in clear polycarbonate buckets (32 cm diameter and 38 cm high, Cambro) with the floor covered by bedding. Food, water and nest material were also added. The video rate captured by an infrared-sensitive USB camera was 11–13 frames/s providing a movement resolution of ∼76–90 ms. The iSpy software was used to determine the motion of the animals through a frame-by-frame subtraction of successive video images. The threshold for movement detection of the software was set to 80 pixels.

### Analysis of *in vivo* LFP recordings

All data analyses were carried out using custom-written procedures in IgorPro 8.0 (Wavemetrics) using its built-in functions for root mean squared (RMS) measurement, FIR filtering, fast Fourier transform (FFT), Hilbert amplitude, etc. The rest of the analyses such as Morlet wavelet transform, spectral peak detection, phase–amplitude coupling (PAC) and frequency–amplitude coupling (FAC) were done using procedures written in Igor 8.0 based on published methods. First, using a FIR filter [using at least 401 coefficients or with the number of coefficients determined as = int(50/(22*(HF-LF)/SR)], where int is the integer part, 50 is the cut-off of the filter in dB, HF and LF are the high and low frequencies, respectively, of the bandpass and SR is the sampling rate (2048 s^−1^). We bandpass filtered the raw recordings, at 0.5–4 Hz for δ, 5–12 Hz for θ and 30–120 Hz for γ oscillations. This digital FIR filtering did not change the phase of the oscillations (Supplementary Fig. 3A). The bandpass-filtered traces were then used for calculating the RMS values for the analyses. For assessing the connectivity between the two recording sites for δ oscillations during non-REM (NREM) sleep or wheel running, we calculated the intersite phase clustering (ISPC), a better term for the phase-locking value.^56^ It was calculated as $\mathrm{ISPC}=\;|n^{-1}\sum_{t=1}^{n}e^{i(\phi_{xt}-\phi_{yt})}|$ where *n* is the number of time points, *ϕ_x_* and *ϕ_y_* are the phase angles calculated from the Hilbert transforms at the vCA1 and mPFC electrode locations, respectively.

### Vigilance state determinations

The RMS values for the γ and δ bandpass filtered recordings were calculated in 8 s long epochs shifted by 0.5 or 1 s steps as previously described.^57^ In brief, the RMS(γ)/RMS(δ) ratios were calculated on a point-by-point basis and plotted for 12 h recording periods corresponding to the L or the D cycles. A histogram of the RMS(γ)/RMS(δ) ratios was plotted for an entire 12 h (43 200 s) recording period. The histograms were the best fit by two Gaussian distributions. The Gaussian with the lowest mean and variance, indicating a low RMS(γ)/RMS(δ) ratio, was considered as the starting point for identifying NREM periods. To further refine the NREM periods, we used the probabilities relative to the SD of a normal distribution. In such distributions, the probability of values lying between the mean and 1 × SD from the mean is 68.27%, between the mean and 2 × SD from the mean is 95.45% and between the mean and 3 × SD from the mean is 99.73%. Thus, for each 8 s segment of the RMS(γ)/RMS(δ) ratios, we calculated the probability of the given segment belonging to the Gaussian with the lowest mean in the distribution of RMS(γ)/RMS(δ) ratios. The decision about a given segment belonging (or not) to NREM was made on the basis of averaging probabilities in 8 s segments both before and after the 1 s segment of the RMS(γ)/RMS(δ) ratio. A continuous probability value as a function of time was calculated using three different probability levels assigned point-by-point (1 s epochs) for values of the RMS(γ)/RMS(δ) ratios (*G*/*D*) in the lowest mean Gaussian distribution with a standard deviation of SD as fitted using the Igor Pro 8 Gauss curve-fitting procedure. The probabilities for the 1 s *G*/*D* segments were assigned as follows: a value of 1, if *G*/*D* ≤ mean+1 × SD; a value of 0.397 [i.e. (95.45 − 68.27)/68.27], if mean + SD < *G*/*D* ≤ mean+2 × SD and a value of 0.067 [i.e. (99.73 − 95.45)/68.27], if mean+2 × SD < *G*/*D* ≤ mean+3 × SD. From this probability function, a binary NREM classification was constructed by assigning a value of 0 if the average of the probability values was <0.5 during both the 8 s prior and 8 s after the 1 s epoch in question. If the average of the probabilities during the two 8 s segments was ≥0.5, the value assigned to the binary NREM classifier was 1. Once the NREM periods were thus identified, we calculated the RMS(θ)/RMS(δ) ratios of the θ and δ bandwidth-filtered recordings of the segments lying outside the already identified NREM periods. These were the segments to be subsequently classified as REM or AWAKE, as follows. First, we constructed all-point histograms of the RMS(θ)/RMS(δ) ratios of these segments, as θ/δ ratios are known to be highest during REM sleep in rodents.^58^ These histograms could be best fitted by two Gaussians. We considered the intersection of the two Gaussian distributions (the point where the probabilities of belonging to either of the distributions are equal) to define the objective threshold for the RMS(θ)/RMS(δ) ratios separating the two distributions. The initial classification between REM and AWAKE was done based on the two RMS(θ)/RMS(δ) distributions. The 1 s epochs were classified as REM if their RMS(θ)/RMS(δ) was above the objective threshold (i.e. it fell into the Gaussian with the larger mean), and if the segment was flanked at least on one side by an already identified NREM period. The activity (Act) levels of the mouse as obtained from the frame-by-frame subtraction of the video recordings were used to further refine the distinction between REM and AWAKE. The Act was analysed separately during the established NREM periods, when the animal was resting, and separately during REM or AWAKE periods (outside of the NREM periods). The REM classified segments based on the RMS(θ)/RMS(δ) distributions were reclassified as follows: where the Act was greater than the median Act (during REM or AWAKE), the REM segments were reclassified as AWAKE and original AWAKE segments where the Act was lower than the median Act (during NREM) were reclassified as REM. Where transitions of AWAKE to REM occurred, the REM was also reclassified as AWAKE. The fractions of NREM, REM and AWAKE periods during every hour were expressed as % total and were entered into a tab-delimited text file to be later used for analyses. Our vigilance state distributions during 12 or 24 h periods (e.g. Table 2) are in agreement with recently published findings in mice^59^ where more sophisticated methods, including multisite LFP recordings and EMG were used. Moreover, our finding that γ oscillation frequencies are higher during REM periods than during AWAKE regardless of SAL or VCD treatment (Supplementary Fig. 4), as has been shown in mice^59^ and monkeys,^60^ also indicates our correct classification of REM periods.

### Determination of spectral peaks for θ and γ oscillations

Spectral peaks on the FFTs of the filtered or raw signals within a frequency band of relevant oscillations in the brain are considered to be the meaningful relative to the ‘pink’ 1/*f^α^* spectrum, where *f* is the frequency of the FFT and *α* is a scaling factor, decline of power in the frequency spectra of LFP.^25,61,62^ We have previously described a procedure to determine the true spectral peaks of the θ and γ oscillations.^63^ Briefly, over the 12 h long raw LFP recording, we used an 8 s long sliding window in steps of 1 s (i.e. 7 s overlaps) to calculate the FFT in each of the 8 s segments. We then smoothed the power of the FFT between the frequency intervals of the θ (5–12 Hz) and γ (30–120 Hz) oscillations. Next, we drew a line connecting the boundary frequencies of the θ and γ oscillations. The frequency and the magnitude of the spectral peaks were calculated relative to this line drawn on the log–log plots of FFT magnitude versus frequency i.e. ‘whitening’.^62^ The hourly average spectral peak values determined during each segment were calculated for both θ and γ oscillations. We discarded the magnitude data as these values are biased by electrode impedance and location. However, the spectral peak frequencies are accurate, and thus, we could compare numerous hourly events over long recording periods of up to several weeks. During each hour of recording, the spectral peaks were separately averaged for the three vigilance states NREM, REM and AWAKE.

### Determination of FAC and PAC of θ and γ oscillations

After isolating periods with spectral peaks as described above, we identified 8 s long epochs when both θ and γ oscillations showed significant spectral peaks during AWAKE and REM periods. We used published methods^56,64–66^ to calculate the modulation indices of γ oscillations by θ oscillation frequency or phase. We used two methods to determine the coupling of the θ oscillation phase to γ oscillation amplitude. In the first method, we started by isolating the individual θ cycles from the θ-filtered traces during the 8 s epochs yielding ∼50–70 cycles in total. Then, each θ cycle was divided into equal time segments each corresponding to π/9 radians of the given θ cycle. The γ bandwidth-filtered traces were then aligned with these time segments and their peak-to-peak amplitudes and frequencies were measured. The median values of these amplitudes were entered into a matrix in the corresponding gamma frequency row binned by 10 Hz from 30 to 120 Hz. The matrix was complete when all the θ cycles during the 8 s epoch were analysed. The second method used the Hilbert transform of the θ bandwidth-filtered signal over the 8 s epoch to calculate the continuous phase of the θ oscillations. Over each phase of the θ cycle thus obtained, the raw signals were filtered in 10 Hz bins between 30 and 120 Hz (14 bins in total) and the amplitude histograms of these band-filtered signals were plotted over each θ phase. The two methods yielded nearly identical PAC results, but we decided to show the graphs obtained by the first method. Once the various modulation matrices were obtained for at least 10 different 8 s periods during the same vigilance state, we averaged the matrices preferably recorded during the same hour. The resulting averages were converted to a *Z*-scale (Fig. 3A and B) and were smoothed by a 2D spline algorithm (ImageInterpolate function of Igor 8.0).

### SPW-R and sleep spindle detection

SPW-R and sleep spindles were detected during NREM periods and quantified simultaneously using a custom Igor procedure. To detect SPW-R, vCA1 hippocampal LFP recordings were first FIR bandpass filtered in the ripple frequency range (120–250 Hz). This bandpass signal was squared and then square rooted to rectify the signal and SPW-R events were identified from the RMS envelope by identifying epochs with a *z*-score of ≥ 3.^67^ The beginning and the end of the events were marked when the *z*-scores returned to 0. When the averages of the SPW portion of the events were compared with the two baseline portions of the recordings of equal time of the duration of the detected SPW-R event, the differences were within one SD of these segments (Supplementary Fig. 3B and C). This was taken as evidence for the recordings of the SPW-R to originate from within, or in close proximity to the vCA1 PC layer. The same detection procedure was set to detect spindles from mPFC LFP recordings. mPFC traces were bandpass filtered between 10 and 30 Hz. The threshold for spindle event detection was set at 5 SD above the baseline of the normalized and rectified signal envelope. The minimum duration for spindle events was set at 5 ms, but the average duration of the spindles was about 10 × this value. The burstiness and memory of SPW-R events were calculated according to the formula previously described for the analysis of sIPSCs.^68^ A value of 1 for burstiness indicates a ‘bursty’ occurrence of events (i.e. when SD is very large) while a value closer to −1 (i.e. when SD is very low) indicates a highly regular SPW-R distribution. A value of 0 indicates that the inter-event interval (IEI) distributions follow a random Poisson process. A positive memory indicates the propensity for short IEIs to be followed by short IEIs and long IEIs by long IEIs. A negative value indicates that short IEIs are more likely to be followed by long IEIs and long IEIs tend to be followed by shorts IEIs. A value close to 0 reflects an absence of memory in the system generating the SPW-Rs.

### Running on the wheel and its detection

After at least 10 days following the beginning of the *in vivo* recordings (i.e. 12.5–13.5 weeks after the last day of VCD administration; Supplementary Fig. 1), a running wheel consisting of a 5 inch (12.7 cm) diameter tilted (∼30°) exercise saucer (Pet Champion, Walmart) was introduced into the recording/home cage of the mouse at the same time of the day (5 PM), just 1 h before the onset of the dark cycle. The running wheels were left in the cages for ∼1.5 weeks, so these experiments were concluded 14–15 weeks after the last day of VCD injections. The running wheel was previously disinfected with 70% ethanol, and stripes of infrared light-reflecting tape were applied to its lower surface to enhance the visibility of its movement when the mouse was running on it (Fig. 4A). This was necessary to detect the running of an otherwise stationary appearing running mouse as large movements on the video capture software that calculates movement as pixel-by-pixel differences between successive video frames. The movements of the mouse including the motion of the running wheel were captured using a USB infrared camera and iSpy software. Due to the large movement artefacts caused by the stripes moving with the running wheel, the movement detection threshold could be set to a high level to detect just the movement of the running wheel. The automated detection was routinely checked against the scoring of an unbiased observer and the running parameters were adjusted until at least a 99% correspondence was achieved by the software with the visually scored running periods.

### Statistical analyses

Unless indicated otherwise, most statistics are reported as mean ± standard deviation (SD). Most of our data were compared using the non-parametric Wilcoxon–Mann–Whitney (MWW) two-tailed rank-sum test for unpaired data and the Wilcoxon two-tailed signed-rank test for paired data. For the comparison of multiple groups, we first tested for significance using the Kruskal–Wallis (KW) non-parametric analysis of variance test followed by the non-parametric multiple comparison Dunn–Holland–Wolfe test allowing for unequal sample sizes. Cumulative probability distributions were compared using the Kolmogorov–Smirnov (KS) test. Significance levels were set at *P* < 0.05, except for the KS tests where it was set to *P* < 0.001. Whenever possible, the exact probability values are given. All statistics were performed using the built-in functions of Igor Pro 8.0 (Wavemetrics). Since large sample sizes can produce significant statistical *P*-values even in the absence of meaningful changes in the means under scrutiny,^69^ we also calculated the effect size according to Cohen’s *d* = ABS(*M*_1_ − *M*_2_)/SQRT((SD_1_^2^ + SD_2_^2^)/2) where *M* is the mean and SD is the standard deviation of the respective distributions (1 and 2). According to the scale, the *d* values define the effects sizes as: small (*d*≤0.2); medium (*d* = 0.5), large (*d* = 0.8), and very large (*d*≥1.2).

### Data and code availability

The data sets and code generated during this study will be made available at UCLA Dataverse (https://dataverse.ucla.edu/).

## Results

### Circadian changes in θ and γ oscillations

**Fig. 1:**
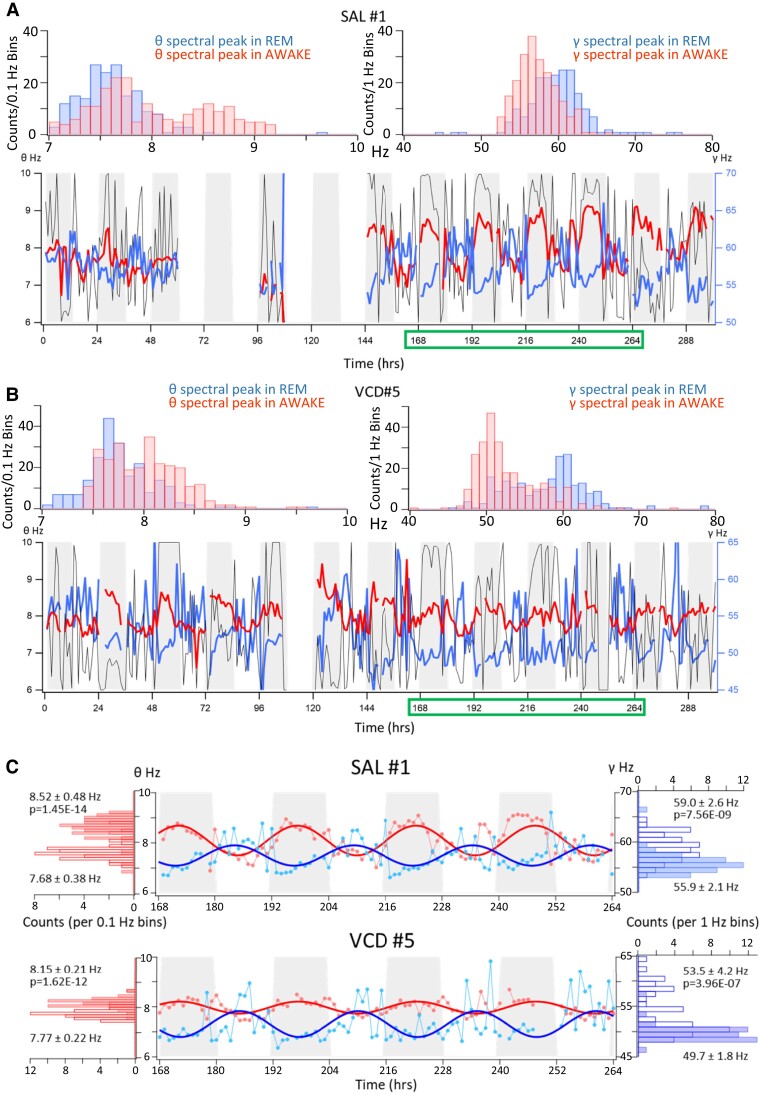
**Representative θ and γ frequency spectral peaks measured over 1 h intervals recorded over several days in a SAL and a VCD-injected mouse.** (**A**) *Top panels*: Histograms of the average θ and γ hourly spectral peaks during awake and REM periods from a SAL-injected mouse. *Bottom panel*: Average hourly values of the spectral peak frequencies for the θ (*red*) and γ (*blue*) oscillations plotted over 13 days of recordings. Grey bars indicate the D cycles, and white bars indicate the L cycles. Dark lines indicate the animal movements on an arbitrary scale as calculated from the pixel-by-pixel differences from the video recordings. Note that some of the recordings are missing. (**B**) Same as in **A**, but for a VCD-injected mouse. Note the lower boundaries but the same *y*-scaling of the γ oscillations compared with the SAL mouse, also illustrating the lowered frequency of these oscillations in the VCD-injected mice. The green boxes on the *x*-axis labels in **A** and **B** indicate the four consecutive days plotted in more detail in **C**. (**C**) Four consecutive recording days depicted from **A** and **B** of the two mice. The hourly average spectral peak frequencies are clearly illustrated as round symbols for θ (*red*) and γ (*blue*) oscillations. Fitted lines are sine waves with 24 h repeat periods. The histograms on the sides indicate the distribution of the hourly average spectral peak frequencies of θ (*red*) and γ (*blue*) oscillations. The open bars refer to the L cycles while the filled bars refer to the D cycles. The numbers illustrate the mean ± SD of the distributions and the *P*-values were obtained by comparing the means using the unpaired *t*-tests for *n* = 46–48 hourly values. For the four comparisons, Cohen’s *d* values range from 1.16 to 1.92.

To shed some light on various features of the menopausal brain state, we sought to characterize the brain activity of AOF mice in the peri-menopause stage (Supplementary Fig. 1) using intracranial electrophysiological recordings. More specifically, as shown in Supplementary Fig. 2, we recorded LFPs ipsilaterally in the brain regions of ventral hippocampal CA1 (vCA1) and medial prefrontal cortex (mPFC). We focused on these two regions based on the involvement of vCA1 in the control of emotional and anxious behaviours^70–72^ and on the existing underlying projections from vCA1 to the prelimbic subdivision of mPFC involved in anxiety.^73^ The location of the recordings in or very near the vCA1 PC layer was ascertained by the minimal deflections compared with the baseline of the sharp wave part of the detected SPW-R in all of our recordings (see Materials and methods). We were able to record continuously for several days while the mice moved freely in their home cages. During every hour of recording, we determined the average values of the spectral peaks corresponding to the θ (5–12 Hz) and γ (30–120 Hz) oscillations.^63^ This long-term approach revealed novel circadian variations in the hourly spectral peaks of the two oscillations that were out of phase with each other. As shown for representative SAL-injected (SAL; Fig. 1A) and VCD-injected (VCD; Fig. 1B) mice, the peak of the hourly theta frequency was observed in the middle of the dark cycle, when the frequency of the gamma peak was at its lowest. The hourly γ spectral peak reached its highest value during the light phase of the diurnal cycle, when the θ frequency was at its lowest (Fig. 1C). The regular cycling of the θ and γ oscillations was not evident during the first few days of the recordings after the animal has been placed in its home/recording cage. Apparently, as the animals needed to acclimatize for 4–7 days for the diurnal cycles to develop (Fig. 1A and B). Conceivably, this is the reason why such circadian changes in the frequencies of the two types of oscillations were not previously described.

### Changes in θ and γ oscillation frequencies in menopausal mice

**Fig. 2:**
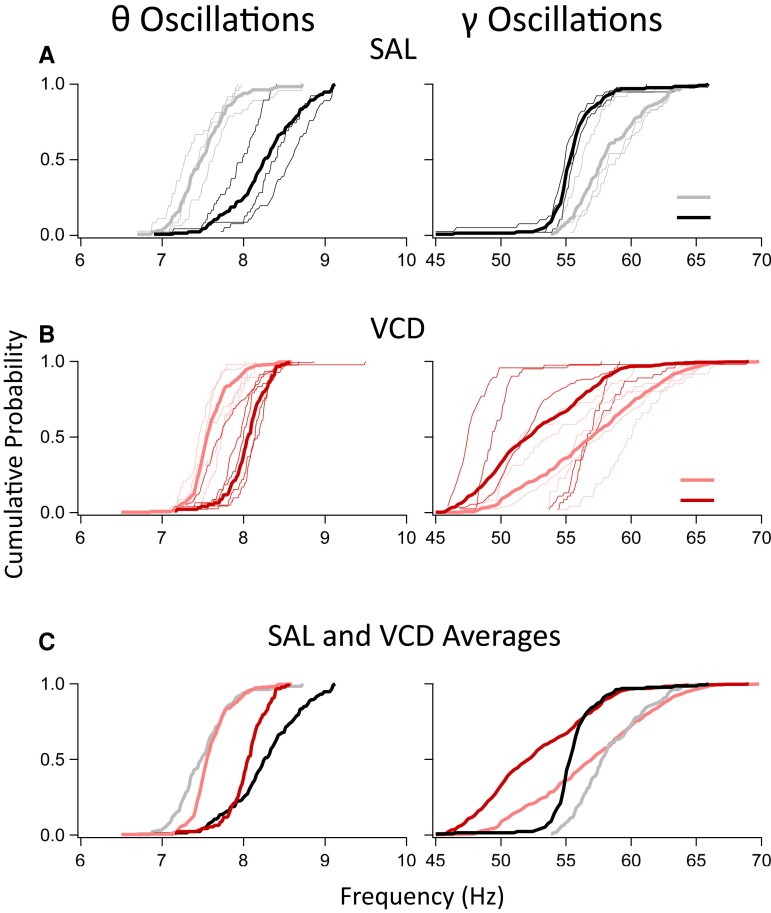
**Distribution of the cumulative probabilities of θ and γ frequency spectral peaks measured over periods of AWAKE states in SAL- and VCD-injected mice during the L or D phases of the diurnal cycle.** (**A**) Distribution of the oscillation frequencies during SAL (*n* = 3) mice. The distributions in the individual mice are shown in thin lines while thick solid lines indicate the averaged distributions. The darker colours (*black*) are during the D phases of the cycle while the lighter colours (*grey*) are during the L phases. The θ oscillations have significantly lower frequencies during the L phase (KS test, *P* < 0.0001) while the opposite is true for γ oscillations which have higher frequencies during the L phase (KS test, *P* < 0.0001). (**B**) Same as in **A**, except for VCD-treated mice (*n* = 5). Although the distribution of frequencies are more widespread, the same findings are present in the VCD mice, i.e. the θ oscillations have significantly lower frequencies during the L phase (KS test, *P* < 0.0001) while the γ oscillations increase their frequencies during the L phase (KS test, *P* < 0.0001). (**C**) Comparison of the average cumulative probability distributions of oscillation frequencies between SAL and VCD mice. For both θ and γ oscillations and both L and D phases of the diurnal cycle, the oscillation frequencies are significantly higher in the SAL mice (KS test, *P* < 0.0001). The comparisons of the θ and γ oscillation frequencies during the REM periods can be found in Supplementary Fig. 4.

In addition to the diurnal cycling of the θ and γ oscillations, we observed statistically significant differences in the mean θ and γ frequencies between the VCD and SAL-injected control mice. During AWAKE periods, the cumulative probability distributions of the frequencies of both hippocampal θ and γ oscillations (Fig. 2A) in individual SAL mice (*n* = 3), as well as their averages, show considerable light–dark (L–D) cycle variations with θ frequencies higher during the dark and γ frequencies higher during the light phases of the cycle. A similar distribution was observed in VCD-injected mice (*n* = 5), albeit with a larger variability (Fig. 2B). Figure 2C shows the differences in the θ and γ frequencies using the averages of the cumulative frequency distributions. The SAL θ frequencies are lower than those in VCD during the L phases of the cycle but are higher than VCD during the D phase. The γ frequencies in SAL animals were higher than those in VCD mice during both phases of the diurnal cycle, evidenced by a shift towards higher values at the low γ frequency range. Interestingly, during REM sleep periods, the cumulative distributions of the θ and γ frequencies did not significantly differ between SAL and VCD mice (Supplementary Fig. 4A). The REM θ oscillation frequencies were closer to the lower frequencies found during AWAKE periods during the L phases of the cycle (Supplementary Fig. 4B). However, the γ oscillation frequencies during REM in both SAL and VCD mice significantly surpassed even the large γ frequencies recorded during the L cycles of the AWAKE periods (Supplementary Fig. 4B). This is in line with recordings in mice^59^ and monkeys^60^ where the largest γ frequencies are recorded during REM sleep. Since θ and γ oscillation amplitudes, frequencies and their phase amplitude coupling is involved in numerous aspects of cognitive, memory and learning processes,^19,65,74–76^ the significant changes in θ and γ oscillation frequencies in VCD mice compared with SAL controls might be a sign of distinct functional brain states and network events that have yet to be discovered in the AOF menopause model.

### Reversed PAC between θ and γ oscillations in menopausal mice

The synchronized activity of neuronal populations in the brain that results in neuronal oscillations has been associated with a variety of cognitive processes. Oscillations of different frequencies are not independent, but they interact and modulate each other. One form of cross-frequency coupling that enables the integration of information from multiple neuronal ensembles across different temporal and regional levels is the FAC while another is the PAC, where the frequency or the phase of the lower frequency oscillation regulates the amplitude of higher frequency oscillations. Notably, FAC and PAC between θ and γ oscillations have been implicated in information processing in visual perception, working memory, attention, and signal detection.^64,77–80^ A disorganization of PAC dynamics has been associated with several neurological and psychiatric disorders.^81–84^

**Fig. 3:**
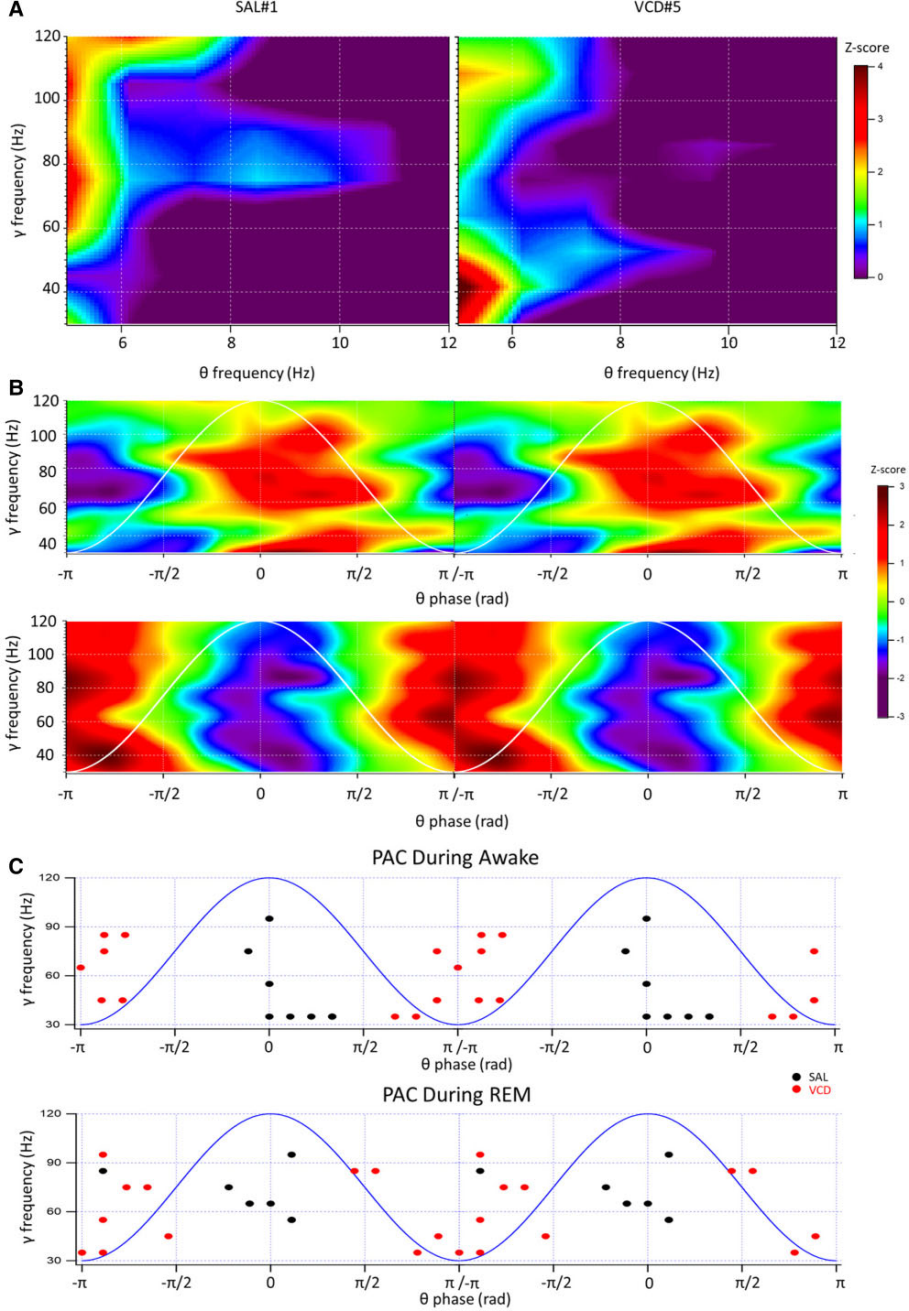
**Analysis of the θ–γ modulation index (MI) and PAC coupling**. (**A**) MI matrix for each θ–γ frequency pair shows by how much γ amplitude distribution for each θ frequency bin deviates from an uniform distribution (Kullback–Leibler distance between two distributions).^65^ The MI is independent of the amplitude and phase locations, it just shows coupling strength (colour scale indicates the Z-score of the MI). The two panels are from two different mice SAL#1 (*left panel*) and VCD#4 (*right panel*). Each panel is the average of at least 10 individual MI matrices calculated from 8 s epochs taken from the same 12 h AWAKE periods in each of the two animals. (**B**) PAC in the same two mice. *Top panel*: SAL#1 mouse, *bottom panel*: VCD#5 mouse. The colour-scale plots show the *Z*-scores of PAC of γ oscillation amplitude to the phase of the θ oscillations with the 0 radian point being at the peak of the θ wave. Two repeat cycles of θ oscillations (*white*) with their peaks and descending phases coupled to the peak γ power. As in **A**, at least 10 matrices of PAC have been averaged from 8 s epochs during the same AWAKE periods. (**C**) *Upper panel*: PAC in randomly selected seven segments from *n* = 3 SAL mice during the AWAKE state (*blue*). The points represent the coordinates of the maximum coupling in an averaged non-Z-score converted matrix of at least 10 individual analyses of 8 s epochs. The *red* symbols are 10 randomly selected segments from *n* = 4 VCD-treated mice plotted similar to the SAL mice. Note the correlation of the peak coupling regardless of γ oscillation frequencies with the peak and the descending phase of the θ oscillations in SAL animals, and the diametrically opposite coupling with the trough of the θ oscillations in VCD animals. *Bottom panel*: The same characteristic patterns of PAC can be found during randomly selected REM periods (*n* = 6 for SAL, and *n* = 11 for VCD).

As expected from the lower γ oscillation frequencies, the θ−γ FAC in VCD mice was shifted to lower γ frequencies, but the θ oscillation frequencies where coupling was maximal remained largely unaltered (Fig. 3A). The θ−γ PAC showed a more conspicuous pattern. Most notably, in contrast to the SAL-injected controls, the VCD mice displayed a striking reversal of the PAC. As shown by previous studies,^55,64,65^ the highest coupling ratios between γ oscillation amplitude and the phase of θ oscillations occurs at the peak, or right after the peak, during the descending phase of the θ cycle. This was also seen in our SAL-injected mice (Fig. 3B), whereas in the VCD-injected mice, the highest amplitude γ oscillations were coupled to the troughs (as detected in or very near the vCA1 PC layer) of the θ oscillations (Fig. 3B). This pattern seen during the AWAKE state also persisted during the REM sleep stage in all of the animals examined (Fig. 3C), when θ − γ PAC is also prominent.^55,59^ The apparent disruption in the hierarchy of the cross-frequency modulation between θ and γ oscillations after AOF might be instrumental for the understanding of the neural mechanisms underlying the menopausal brain state.

### Voluntary wheel running and δ oscillations during running and NREM sleep

**Fig. 4:**
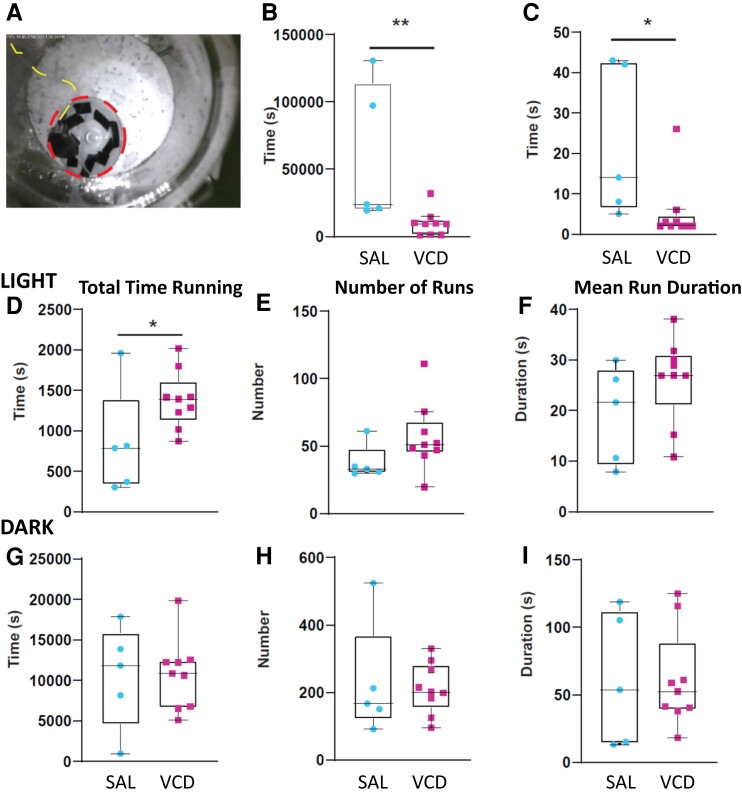
**The performance of VCD mice on the running wheel is associated with anxiety-related behaviour.** (**A**) Example of a mouse engaging in voluntary running activity on a running wheel inside its home/recording cage. The circumference of the wheel is highlighted by the red dashed line and the recording cable is indicated by the yellow dashed line. (**B**) Plot showing the time elapsed until the first run since the introduction of the running wheel in the cage. The VCD mice engage faster than SAL mice in running activity on the wheel (*P* = 0.007, Cohen’s *d* = 1.29). (**C**) However, the first runs of the VCD mice are shorter in duration (*P* = 0.007, Cohen’s *d* = 1.19). (**D**–**I**) Monitoring the running activity of the mice over the course of several days, it is revealed that during the L cycle, the VCD mice have a longer total time running (**D**) (*P* = 0.05, Cohen’s *d* = 1.01) resulting from an elevated number of runs (**E**) (*P* > 0.05, Cohen’s *d* = 0.91) and an increase in the mean run duration (**F**) (*P* > 0.05, Cohen’s *d* = 0.77), in comparison to their SAL counterparts. In sharp contrast, during the dark cycle where mice are much more active, the running pattern of VCD mice was not different from that of SAL mice (**G–I**). Box and whisker plots show the range of individual data points, with the interquartile spread as the box, the median as the line bisecting the box and Tukey style whiskers. All data were generated from *n* = 5 SAL and *n* = 8 VCD mice. All statistics were done using the MWW test.

Voluntary wheel running is an approach to study the progress/adaptation of physical exercise in mice which changes in several disease models and with ageing.^85^ In addition, numerous studies emphasized that wheel-running activity in captive rodents is in and of itself a complex behaviour also related to anxiety- and depression-related states.^86^ Because of its relationship to energy balance, voluntary wheel running in mice is inexorably linked to stress response, mood and reward. Given the association of human menopause with changes in mood and an increase in anxiety and depression, we decided to exploit this behavioural paradigm VCD- and SAL-treated mice. Moreover, given the chance, voluntary wheel running is practiced by mice living in the wild without the expectation of any reward, indicating that this elective behaviour is a natural need for the well-being of mice, regardless of whether in captivity or not.^87^ Therefore, we wanted to examine the potential differences between control and AOF mice, as only one previous study addressed this behaviour in mice injected with VCD at a much younger age (2 months of age).^88^ In the home cage/recording chamber of the animals an hour before the lights went off (5 PM), we introduced a slanted running wheel^85^ with stripes mounted on its bottom surface for better video recognition (Fig. 4A) to compare their voluntary wheel running behaviour. At the same time, we also recorded the corresponding LFP activity in the vCA1 and mPFC of some of the SAL- and VCD-injected mice. Following introduction of the wheel into the home cage, we tested the latency to the first attempt of running and the duration of the first run. After the introduction of the wheel into the home cage, VCD-injected mice started running on the wheel much sooner (mean ± SD: 2.77 ± 0.87 h, *n* = 9) than their SAL-injected counterparts (162.21 ± 6.47 h, *n* = 5; *P* = 0.007, Cohen’s *d* = 1.29; MWW test; Fig. 4B), albeit for a much shorter initial duration (VCD: 5.3 ± 2.6 s; SAL: 22.4 ± 8.3 s; *P* = 0.007, MWW test; Cohen’s *d* = 1.19; Fig. 4C). The fact that VCD-injected mice mounted the wheel and ran after <3 h following its placement in their cages, while it took the SAL-injected mice almost a week to do the same, indicates distinct behavioural differences between the two groups. We continued to monitor the wheel running activity of the mice over a period of 2 weeks. As expected, during this time, voluntary wheel running was predominantly a D cycle activity with the mice in both groups running on average about 10 times longer than during the L cycle (Fig. 4D and G). However, the total running time of VCD mice during the L cycle was significantly more than that of SAL mice (VCD: 1384.2 ± 118.2 s; SAL: 845.9 ± 297.1 s; *P* = 0.05, MWW test; Cohen’s *d* = 1.01, Fig. 4D). This was due to the compound effects of an increased number of runs (Fig. 4E) and of the mean run duration (Fig. 4F) in the VCD mice, although individually none of these parameters were significantly different in the two groups. There were no significant differences in the total running time (Fig. 4G), number of runs (Fig. 4H) and mean run duration (Fig. 4I) in the D phase of the cycle between SAL and VCD mice.

Interestingly, in both brain regions (vCA1 and the infralimbic cortex of the mPFC; Supplementary Fig. 2), we observed the occurrence of slow oscillations in the δ frequency range (0.5–4 Hz) while mice engaged in voluntary running on the wheel, along with θ and γ frequency oscillations that normally occur during active locomotor behaviour. Such δ oscillations were recently observed when rats ran on a treadmill to obtain a water reward.^89^ Our findings further demonstrate that δ oscillations also occur when mice engage in spontaneous non forced running activity without any operant conditioning. In addition, as expected, δ oscillations were also present during NREM sleep. We next assessed whether the AOF induces any changes in these slow waves. We analysed in parallel in SAL and VCD-injected mice the δ oscillations in 0.5–4 Hz filtered randomly selected 10 and 20 s long epochs during wheel running and while mice were sleeping. The amplitudes of cortical δ oscillations were significantly larger during running periods than during sleep in both control and VCD-injected mice, but only in AOF mice did this finding hold for the hippocampus (Table 1). Our analysis also showed in both control and AOF mice, the frequencies of δ oscillations in the hippocampus and mPFC were significantly lower during running compared with sleep periods. Most notably, in both control and AOF mice, there is a higher correlation between hippocampal and mPFC δ oscillations during running than during sleep (Table 1). Moreover, in VCD-treated mice, the correlation of δ oscillations between the two brain regions during sleep is significantly lower than in SAL-treated mice (*P*= 7.584e−5, MWW test), while the treatment had no effect on the synchrony during running (*P* = 0.645, MWW test).

**Table 1:** Characteristics of δ oscillations and their correlation between the vCA1 mPFC during voluntary wheel running (RUN) and NREM sleep (SLEEP) in SAL and VCD-treated mice

*n*	vCA1				mPFC			
	SAL		VCD		SAL		VCD	
	RUN	SLEEP	RUN	SLEEP	RUN	SLEEP	RUN	SLEEP
	15	15	20	20	15	15	20	20
Peak-to-peak amplitude (mV)								
Mean	0.210	0.135	0.345	0.126	0.216	0.115	0.324	0.105
SD	0.129	0.028	0.219	0.032	0.129	0.011	0.209	0.019
Wilcoxon	0.38940		0.00013		0.01807		0.00026	
Cohen’s *d*	0.80		1.40		1.11		1.47	
Frequency (Hz)								
Mean	2.71	3.10	2.52	3.26	2.56	3.02	2.51	3.10
SD	0.28	0.13	0.48	0.19	0.38	0.22	0.46	0.20
Wilcoxon	0.00116		0.00001		0.00061		0.00001	
Cohen’s *d*	1.78		2.01		1.51		1.69	
Correlation: vCA1 v mPFC								
	SAL				VCD			
	RUN		SLEEP		RUN		SLEEP	
Mean	0.891		0.589		0.883		0.365	
SD	0.125		0.147		0.160		0.133	
Wilcoxon	6.10 × 10^−5^				1.91 × 10^−6^			
Cohen’s *d*	10.08732		5.66855		7.79765		3.89144	
WMW *P*			0.645					
Cohen’s *d*			0.05					
WMW *P*					7.58 × 10^−5^			
Cohen’s *d*					1.60			

*n* refers to the number of 10 or 20 s long epochs in the δ frequency range (1–4 Hz) filtered recordings that were randomly selected from three SAL and four VCD mice during the same 12 h periods, on average ∼3 h apart. The correlation refers to the cross-correlation values between the δ frequency-filtered segments recorded during the same 12 h periods in the vCA1 and the mPFC. The *P*-values for the Wilcoxon signed paired rank test (Wilcoxon) between the mean values are indicated. For the vCA1 mPFC, we also calculated the *P*-values using the MWM test (MWM *P*) between the SAL and VCD groups matched by RUN or SLEEP. Below all *P*-values, we also show the Cohen’s *d* values for assessment of the effect size for the same comparisons.

**Fig. 5:**
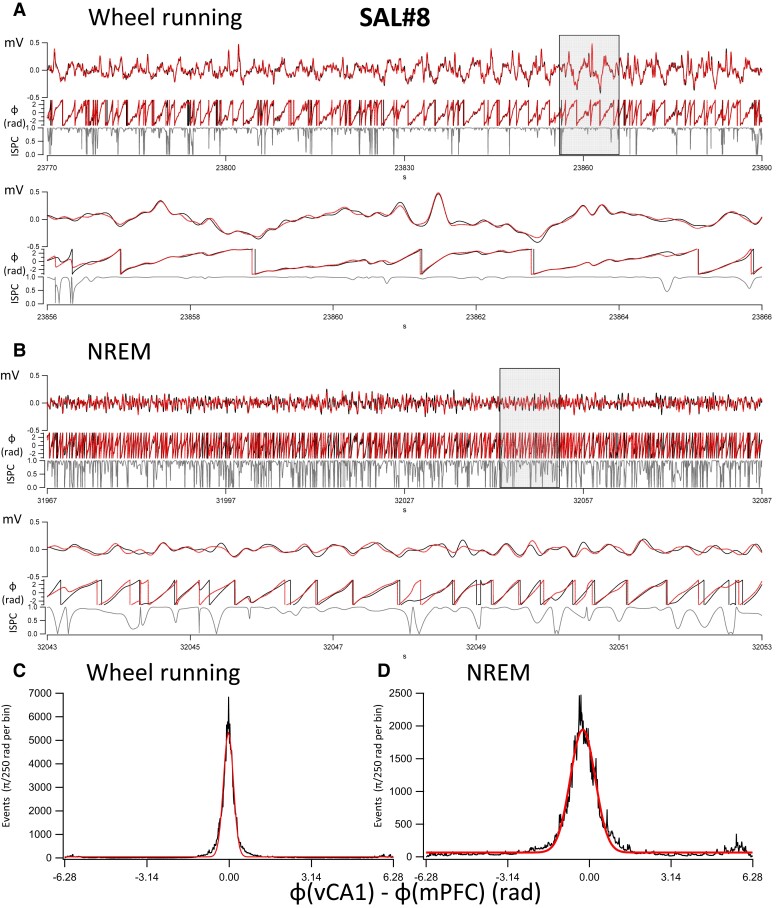
**Synchronization of δ oscillations during wheel running and NREM sleep in a SAL mouse (SAL#8).** (**A**) A 120 s long recording of δ bandwidth-filtered (0.5–4 Hz) recording in the vCA1 (*black*) and mPFC (*red*) during wheel running. The wheel running has been verified in the video recordings for the entire duration of the 120 s segment. The three traces on each panel are from top to bottom: δ bandwidth-filtered trace (*y*-scale: mV), the phase of the oscillations calculated from the Hilbert transform (*y*-scale: phase in radians from −π to π), and the instantaneous ISPC or phase-locking value (*y*-scale: 0–1). The average ISPC value for the entire 120 s epoch was 0.934. The lower panels represent a 10 s long cutout from the upper panels during the period indicated by the grey rectangle. (**B**) Same as in **A**, but during an NREM sleep period. The average ISPC value for the entire 120 s epoch was 0.789. (**C**) Histogram of the phase differences between the vCA1 and mPFC δ oscillations during the 120 s wheel running epoch. The Gaussian fit (*red line*) to the histogram has a mean of −0.031 rad, equivalent to – 2.03 ms (based on the compound average frequencies determined from the phase distributions, i.e. 2.45 Hz, of the δ oscillations in the two regions). (**D**) Histogram of the phase differences between the vCA1 and mPFC δ oscillations during the 120 s wheel running epoch. The Gaussian fit (*red line*) to the histogram has a mean of −0.258 rad, equivalent to – 21.7 ms (based on the compound average frequencies determined from the phase distributions, i.e. 1.9 Hz, of the δ oscillations in the two regions).

**Fig. 6:**
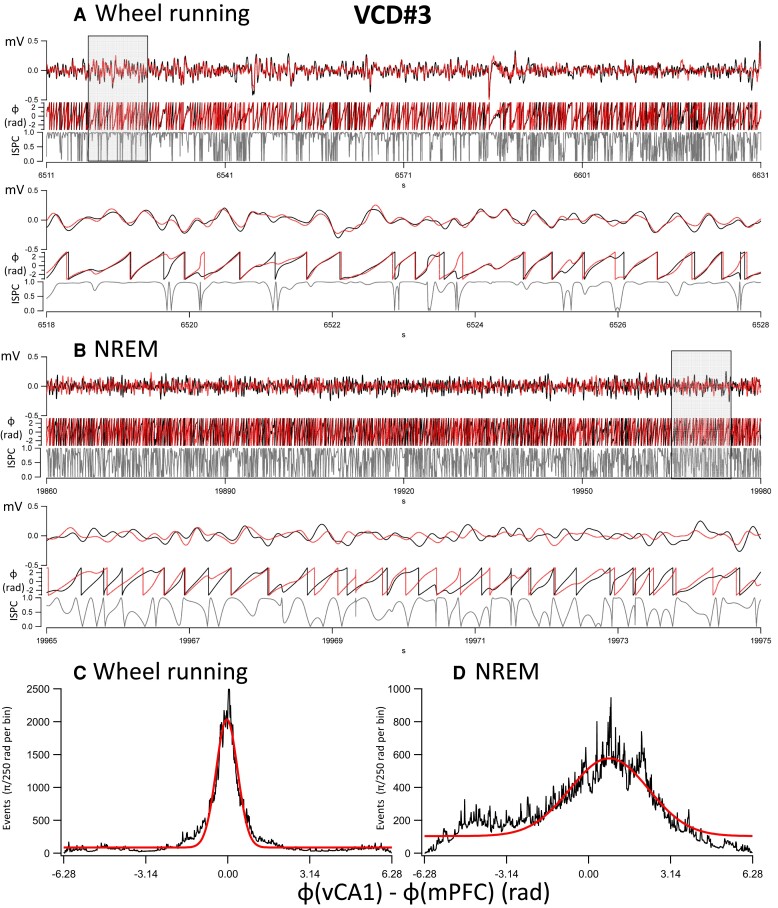
**Synchronization of δ oscillations during wheel running and NREM sleep in a VCD mouse (VCD#3).** (**A**) A 120 s long recording of δ bandwidth-filtered (0.5–4 Hz) recording in the vCA1 (*black*) and mPFC (*red*) during wheel running. The wheel running has been verified in the video recordings for the entire duration of the 120 s segment. The three traces on each panel are from top to bottom: δ bandwidth-filtered trace (*y*-scale: mV), the phase of the oscillations calculated from the Hilbert transform (*y*-scale: phase in radians from −π to π), and the instantaneous ISPC or phase-locking value (*y*-scale: 0–1). The average ISPC value for the entire 120 s epoch was 0.781. The lower panels represent a 10 s long cutout from the upper panels during the period indicated by the grey rectangle. (**B**) Same as in **A**, but during an NREM sleep period. The average ISPC value for the entire 120 s epoch was 0.62. (**C**) Histogram of the phase differences between the vCA1 and mPFC δ oscillations during the 120 s wheel running epoch. The Gaussian fit (*red line*) to the histogram has a mean of −0.035 rad, equivalent to −2.74 ms (based on the compound average frequencies determined from the phase distributions, i.e. 2.03 Hz, of the δ oscillations in the two regions). (**D**) Histogram of the phase differences between the vCA1 and mPFC δ oscillations during the 120 s wheel running epoch. The Gaussian fit (*red line*) to the histogram has a mean of 0.792 rad, equivalent to 64.0 ms (based on the compound average frequencies determined from the phase distributions, i.e. 1.97 Hz, of the δ oscillations in the two regions).

The findings using cross-correlation values between 10 and 20 s long epochs were corroborated in further analyses on 120 s long segments of δ oscillations during NREM sleep and wheel running. Figure 5 shows the analysis in a SAL-injected mouse. The instantaneous ISPC, or phase locking, values were calculated from the phases of the δ oscillations during wheel running (Fig. 5A) and NREM sleep (Fig. 5B). In addition, histograms of the phase differences in radians (also converted to ms based on the average δ oscillation cycle duration) were also calculated during wheel running (Fig. 5C) and NREM sleep (Fig. 5D). Similar analyses are shown in a VCD-injected mouse during wheel running (Fig. 6A and C) and NREM sleep (Fig. 6B and D). The average phase differences between the phases of δ oscillations during wheel running were 0.01 rad (i.e. 0.5 ms) in SAL animals (*n* = 3) and 0.04 rad (i.e. 3.2 ms) in VCD animals (*n* = 5), respectively. This tight difference between the phases of the δ oscillations during wheel running may indicate that the oscillations are volume conducted^56^ to both sites from a common site of origin. The phase difference values were −0.14 rad (i.e. −12.1 ms) and 0.21 rad (i.e. 16.1 ms), in SAL and VCD mice, respectively, during NREM sleep. The significance of sign reversal between these phase differences is not known at this time, but it may be resolved in future studies using multiple site recordings. The average (± SEM) ISPC values during the same 120 s wheel-running epochs were 0.88 ± 0.05 in SAL (*n* = 3) mice and 0.87 ± 0.02 in VCD (*n* = 5) mice, with no significant differences between the groups. In contrast, during NREM sleep, these values were 0.748 ± 0.028 (95% CI: 0.694–0.801) for SAL mice, significantly (MWM, *P* < 0.05) higher than the 0.645 ± 0.012 (95% CI: 0.622–0.668) for the VCD-treated mice. This finding is in line with the data obtained from the cross-correlations between different segments of wheel running and NREM sleep (Table 1), and substantiate the idea that there is a decreased communication between the vCA1 and the mPFC during NREM sleep.

### Hippocampal SPW-Rs and their coupling to mPFC sleep spindles in menopausal mice

**Fig. 7:**
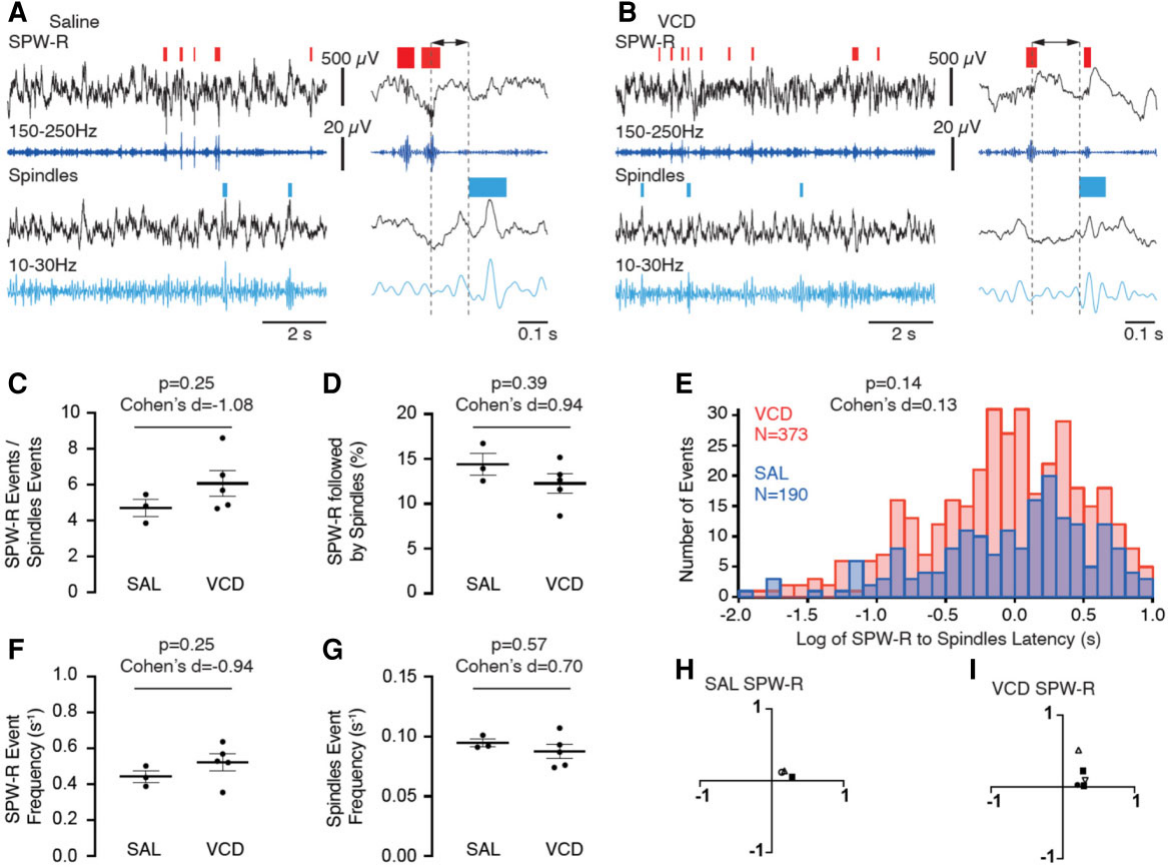
**Temporal organization of SPW-R and their interaction with sleep spindles events during NREM sleep in VCD- and SAL-injected animals.** (**A**) Sample recording of hippocampal and cortical LFP during NREM sleep in a SAL-injected mouse. Hippocampal LFP and matching filtered trace (150–250 Hz) were used to detect SPW-R (*red rectangles*, top traces). Cortical LFP and matching filtered trace (10–30 Hz) were used to detect sleep spindles (*blue rectangles*, lower traces). On the right panel, an enlarged portion of the same recording illustrates the measurement of the SPW-R to spindles latency. (B) Same as in **A**, but for a VCD mouse. (**C**) Group comparison of the SPW-R to spindles events ratios. (**D**) Quantification of the percentage of SPW-R immediately followed by spindles. (**E**) Distribution histograms of SPW-R to spindles latency durations from the SAL (*blue bars*) and VCD groups (*red bars*). (**F**) Quantification of SPW-R event frequency in the two groups. (**G**) Quantification of spindles event frequency in the two groups. (**H**) Distribution of the burstiness (*x*-axis) and memory (*y*-axis) of SPW-R in the SAL group. (**I**) Burstiness and memory analysis for the VCD group. Error bars represent SEM and *P*-values were obtained from MWW test (**C**, **D**, **F** and **G**) or two-tailed *t*-test (**E**). Cohen’s *d* values for effect size are indicated on the top of the graphs.

Proper hippocampal–cortical coupling during NREM sleep is necessary for memory consolidation.^31,40^ To determine whether menopause modified such coupling, we analysed the occurrence of SPW-R and spindles and their interaction in SAL and VCD-injected mice. The SPW-R were detected in the vCA1 recordings during NREM sleep, and spindles were detected in the mPFC. The SPW-R to spindle latencies were determined from the simultaneous vCA1 and mPFC recordings (Fig. 7A and B). During NREM in the vCA1, SPW-R was generated at a higher rate than spindles in the mPFC in both SAL and VCD mice. The average ratios of the number of SPW-R to the number of spindles tended to be higher in the VCD mice when compared with the SAL controls (mean ± SD, SAL: 4.70 ± 0.80, *n* = 3; VCD: 6.07 ± 1.59, *n* = 5; *P* = 0.25, MWW test; Cohen’s *d* = 1.08; Fig. 7C). Consequently, the average fraction of SPW-R immediately followed by spindles was lower in the VCD group. Although the difference between the SAL and VCD groups was not statistically significant (SAL: 14.40 ± 2.11%, *n* = 3; VCD: 12.26 ± 2.40%, *n* = 5; *P* = 0.39, MWW test; Fig. 7D), the Cohen’s *d* value (0.94) represents a ‘large’ effect size. The larger number of SPW-R relative to spindles in the VCD group can alone account for a decrease in the fraction of SPW-R followed by spindles in the AOF mouse model of menopause. However, a change in hippocampal–cortical coupling efficiency during NREM sleep may also stem from a considerable increase in the SPW-R to spindle latencies in the VCD animals compared with SAL. To address this possibility, we analysed the distribution of all SPW-R to spindle latencies in both groups (Fig. 7E). The average latencies showed no significant difference between the two groups (SAL: 2.16 ± 2.98 s, *n* = 190; VCD: 1.79 ± 2.40 s, *n* = 373; *P* = 0.14, two-tailed *t*-test (−1.45); Cohen’s *d* = 0.13). Therefore, an increase in the latencies between SPW-R and spindles cannot account for the tendency observed in the AOF menopause model that a larger proportion of SPW-R are simply not coupled to sleep spindles.

SPW-R is the key contributor to memory consolidation and a change in its properties or temporal patterns of expression may reflect a change in CA3 and CA1 network excitability and/or connectivity. Consistent with the change in SPW-R to spindles ratios induced by AOF, there was a trend towards an increase in the frequency of SPW-R during NREM sleep in VCD animals (SAL: 0.44 ± 0.057 s^−1^, *n* = 3; VCD: 0.52 ± 0.047 s^−1^, *n* = 5; *P* = 0.25, MWW test; Cohen’s *d* = −0.94; Fig. 7F), whereas the frequency of sleep spindles tended to decrease, in the VCD group compared with the SAL animals (SAL: 0.095 ± 0.0055 s-^−1^, *n* = 3; VCD: 0.087 ± 0.0060 s^−1^, *n* = 5; *P* = 0.57, MWW test; Cohen’s *d* = 0.70; Fig. 5G). Importantly, the average ripple frequency of SPW-R measured in individual events was not different between the two groups (SAL: 176.6 ± 20.21 Hz, *n* = 1329; VCD: 177.2 ± 22.41 Hz, *n* =2650, *P* = 0.69, MWW test; Cohen’s *d* = 0.028). The average (±SD) duration of the SPW-R events was 53.4 ± 2.2 ms (95% CI: 50.9–55.9 ms) in the SAL (*n* = 3) group, not significantly different (*P* = 0.675, MWW test) from the values of 61.2 ± 18.1 ms (95% CI: 45.4–77.0 ms) in the VCD (*n* = 5) group. Similarly, the sleep spindle durations were comparable in the two groups (*P* = 0.889, MWW test) at 72.0 ± 8.8 ms (95% CI: 62.1–82.0 ms) in the SAL and 81.7 ± 32.4 ms (95% CI: 53.3–110.1 ms) in the VCD group.

To further characterize the temporal patterns of SPW-R events in SAL and VCD animals, we constructed plots of memory versus burstiness according to our previously published methods for spontaneous synaptic events.^68^ The SPW-R was similarly distributed in both groups, to the notable exception of the SPW-R of one animal in the VCD group (triangle symbols) which were expressed with a higher memory than the SPW-R of any other animal (Fig. 7H and I). The inter-group comparison of the average burstiness and memory showed no significant differences.

### Altered sleep and wake cycles in peri-menopausal mice

Based on the evidence of *de novo* sleep disturbances in women arising during the menopause transition and in the post-menopausal stage,^10–13^ we next explored if there are any potential circadian imbalances in the AWAKE, NREM and REM durations during L and D 12 h cycles in SAL and VCD-treated mice. Although, we did not observe any significant differences in the AWAKE and NREM states between the VCD and the SAL groups during the L and the D cycles, both the SAL and the VCD-treated mice showed statistically significant variations in their time spent in AWAKE and in NREM stages (Table 2). As expected, all mice spent significantly more fraction of time in the AWAKE state during the D cycle than during L. Conversely, the percentage of time spent in the NREM phase during the L cycle was significantly longer than the corresponding NREM time during the D cycle (Table 2). Both these variations are expected, since mice, as nocturnal animals, are much less active during the L cycle. The initial KW non-parametric analysis of variance test showed no significant differences between the REM periods in the four groups (Table 2). Therefore, differences between individual groups were not further analysed in multiple comparisons. However, it is worth pointing out that the Cohen’s *d* value (effect size) indicated a moderate effect for the increase in REM sleep percentage in the D over the L cycle in VCD mice instead of the expected decrease that is present in the SAL group (Table 2). Nevertheless, the mean of 17.57% (95% CI: 17.46–17.68%) of time spent in REM sleep during the D cycle is 1.84-fold larger than the 9.53% (95% CI: 9.42–9.64%) REM time fraction spent in the same cycle by SAL mice (Table 2). This finding may hint to stress-associated events in the AOF menopause mice, since it has been shown that increases in REM are early markers of chronic mild stress response.^90–92^

**Table 2:** Per cent time spent in awake, NREM and REM periods during the light (L) and dark (D) cycles

	AWAKE %				NREM %				REM %			
	SAL		VCD		SAL		VCD		SAL		VCD	
	LIGHT	DARK	LIGHT	DARK	LIGHT	DARK	LIGHT	DARK	LIGHT	DARK	LIGHT	DARK
Mean	38.50	57.03	39.57	55.04	48.33	25.00	52.68	28.61	11.08	9.53	7.09	17.57
SD	30.09	38.03	31.69	36.19	24.34	32.00	29.13	29.39	11.80	21.20	10.26	27.73
SEM	2.17	2.66	1.95	2.18	2.12	2.67	1.93	1.90	1.03	1.77	0.68	1.79
*n*	192	204	264	276	132	144	228	240	132	144	228	240
KW *P*	3.65 × 10^−12^				0				0.134			
DHW *P*	L versus D *P* = 5.52 × 10^−7^		L versus D *P* = 1.11 × 10^−6^		L versus D *P* = 1.45 × 10^−9^		L versus D *P* = 0		N/A		N/A	
Cohen’s *d*	0.54		0.45		0.82		0.82		0.09		0.50	
DHW *P*	LSAL versus LVCD *P* = 0.988				LSAL versus LVCD *P* = 0.535				N/A			
Cohen’s *d*	0.03				0.16				0.36			
DHW *P*	DSAL versus DVCD *P* = 0.922				DSAL versus DVCD *P* = 1				N/A			
Cohen’s *d*	0.05				0.12				0.33			

Note that for both SAL and VCD groups, there are significant circadian variations between awake and NREM percentages. *n* indicates the number of 12 h periods used for the comparisons recorded from three SAL and four VCD mice. The non-parametric analysis of variance Kruskal–Wallis test was first done for the four groups (AWAKE, NREM and REM) and if the *P*-values (KW *P*) were significant, all subgroups (SAL Light, Dark, VCD Light, Dark; abbreviated as LSAL, DSAL, LVCD and DVCD, respectively) within the groups were compared with each other. The KW *P*-value was >0.05 for the REM group, and therefore, no further multiple comparisons were carried out. In the AWAKE and NREM groups, the test used for these multiple comparisons was the Dunn–Holland–Wolfe test that allows for unequal sample sizes. The obtained *P*-values are given (DHW *P*). Below these values, we also included the Cohen’s *d* values for evaluation of the effect size.

Other alterations in *ex vivo* electrophysiology, behaviour [elevated plus maze (EPM) and open field (OF)], plasma FSH levels and bone mass loss are described in the Supplementary material.

## Discussion

We have carried out a comprehensive analysis of the brain oscillatory patterns, behavioural and sleep phase correlates, electrophysiological alterations, hormonal and bone metabolism changes in the AOF mouse model^93^ of human menopause. As shown in Supplementary Fig. 1, where the stages of menopause have been adapted to the staging of mice subjected to AOF,^47,49^ our experiments took place during both stages (peri- and post-menopause) equivalent of the human condition. There are numerous studies assessing potential cognitive impairments and behaviour in this rodent model of human menopause,^3,47,94,95^ but this is the first time a systematic analysis of brain oscillations has been done in the AOF mouse model. This new approach might open new clinical studies for the understanding of the neurological and psychiatric alterations during this stage in women’s lives. Our *in vivo* LFP recordings, sleep analyses, and running wheel behaviour experiments were carried out during the peri-menopausal stage, while the OF, EPM behavioural experiments, as well as the *ex vivo* electrophysiology and bone mass determination were done during the post-menopausal stage. Our major findings can be summarized as follows: (i) we report for the first time precise circadian changes in the hourly average spectral peak frequencies of both θ and γ oscillations that were out of phase with each other. (ii) During peri-menopause, AOF mice had different γ and θ oscillation frequencies at distinct phases of the circadian cycle than SAL-injected mice. (iii) During AWAKE and REM sleep periods, γ oscillation amplitudes were coupled to θ phase as previously reported in SAL-injected mice, but this coupling was diametrically the opposite (i.e. shifted by π radians) in the peri-menopausal mice. (iv) Peri-menopause resulted in a tendency of reduced hippocampal SPW-R to mPFC sleep spindles. (v) Voluntary running on a running wheel caused the emergence of highly synchronous large-amplitude δ frequency oscillations between the vCA1 and mPFC, and although this synchrony during running was not different in VCD mice, it was reduced during NREM sleep in mice during the peri-menopause. (vi) There were only mild behavioural changes in the OF and EPM behaviour during the post-menopausal stage. (vii) Tonic and phasic GABAergic inhibitions appear to be unaltered during the post-menopause. (viii) There is a significant decrease in bone density during the post-menopausal period.

The counterphase circadian alterations in the spectral peak frequencies of θ and γ oscillations in the vCA1 have not been observed before. In rats deprived of daytime cues, it was possible to distinguish changes in place cell activity with a 25 h period over short contiguous recordings^96^ and corresponding small (∼2%) changes in reticular stimulation activated θ oscillation frequencies could be recorded when aligned by the timing of food intake.^97^ Although we did not keep track of the time of food consumption by our mice, in our studies, there was no stimulation of a brain area, the animals were exposed to 12 h L–D cycles, and the proportion of place cells in the ventral hippocampus is smaller than that of cells related to anxiety.^73^ We conclude that the counterphase daily periodic changes in θ and γ oscillation spectral peak frequencies as those shown in Fig. 1C reflect genuine and distinct brain states. Although θ and γ oscillations scale with the animals’ speed, the counterphase peak oscillatory frequencies argue against a movement-related phenomenon. It is more likely that the 5–10% changes in oscillation frequencies result from circadian alterations in the multiple types of neurons involved in the generation of these rhythms.^22,25,98–100^ It is interesting to note that the L–D changes in θ and γ oscillation frequencies were also present during the peri-menopausal period, albeit at slightly higher θ and somewhat lower γ oscillation frequencies, and that the development of the periodicities of these oscillations required an adaptation time to the environment (e.g. Fig. 1A). We propose that the diurnal changes in these key oscillation frequencies have consequences on the establishment of the time of the day of the animals, and thus may be involved in the control of numerous brain functions and their adaptation to L–D cycles in the environment.

The lower γ oscillation frequencies during peri-menopause regardless of the L–D cycle (Fig. 2C) may point to a dysfunctional interneuron–principal cell interaction in the ventral hippocampus particularly involving PV + INs that are critically involved in these oscillations.^20–24^ As we gain more insights into the role of diminished γ oscillations in depression,^101^ and in Alzheimer’s disease,^102^ our findings may provide clues to why women during all three stages of menopause have a significantly increased risk of developing depression^6^ and why Alzheimer’s disease prevalence is two to three times higher in post-menopausal women than in men.^17,18^ Clearly, we need to learn more about the regulation of PV + IN function by ovarian hormones and their neurosteroid derivatives, since these cells express ample amounts of the neurosteroid-sensitive δ subunit-containing GABA_A_ receptors that are in a critical position to regulate γ oscillations.^103,104^ Not only was γ oscillation frequency reduced during peri-menopause, but its FAC and PAC to the θ oscillations were also dramatically changed. While the FAC relationship was commensurate with the reduced γ oscillation frequencies, the PAC showed a diametrically opposite phase coupling both during AWAKE and REM periods compared with age-matched SAL animals. As the PAC of γ and θ oscillations is critical for memory and cognition-related associations^19,27,64,74,80^ the peri-menopausal VCD mice may have cognitive impairments and deficits in working memory that will have to be thoroughly tested in future studies. As with the lowered frequency of γ oscillations, we do not know what causes the reversed PAC of γ and θ oscillations. More detailed future investigations in the AOF model will be required to shed light on potential interneuronal impairments that may be critical for the altered oscillatory events and their coupling.

Our studies of the peri-menopausal period also revealed deficiencies between the vCA1 and mPFC infralimbic cortical coupling. This circuit is critically involved in anxiety^73^ and its impairment in pre-menopause was evident in several of our findings. Although the number of SPW-R occurrences during NREM sleep was increased during peri-menopause, measured by the effect size, the coupling link between the hippocampal SPW-R activity and sleep spindle occurrence in the mPFC was reduced. This hippocampal–cortical coupling is critical for memory consolidation,^31,40^ and its tendency to be reduced in peri-menopause may harbinger the decline in memory and cognition in menopausal women.^46,47^ A lack of vCA1 to inferior limbic mPFC coupling was also evident in the reduced coupling between the slow δ frequency oscillations during NREM sleep in peri-menopausal mice. Interestingly, the tight phase coupling between the two structures of the δ frequency oscillations present during voluntary wheel running were not altered by VCD treatment, and may indicate simultaneous volume conduction to the two structures. In itself, the presence of these high amplitude, low-frequency oscillations during voluntary wheel running is intriguing. Recently, similar hippocampal oscillations were described that gradually developed during rewarded short (15 s) treadmill running episodes in rats,^89^ but to our knowledge, the coherent vCA1 and mPFC δ oscillations during voluntary exercise of the mice on a running wheel have not been previously reported. Since this elective behaviour satisfies a natural need for well-being in mice whether in the wild or in captivity,^87^ it is noteworthy that peri-menopausal mice showed a much shorter latency to mount the wheel and run, and spent more time running during the L periods when there is generally a reduced motor activity in nocturnal animals. These findings may mean that the peri-menopausal mice are more driven to run on the wheel to benefit from a presumably rewarding effect of this activity. At the same time, there were no major differences in the percentage of time the two groups of animals spent awake, in NREM or REM sleep during the corresponding D and L periods.

Due to the considerable length of our *in vivo* recordings from both groups of animals, the behavioural and electrophysiological experiments were shifted to the post-menopausal stage (Supplementary Fig. 1). In general, there were no significant differences between the behaviours of the two groups of mice in the OF and the EPM, but there were large effect sizes in the increased speed (and distance run) and time spent in the periphery by the VCD-treated mice in the OF. Similarly, judged by the effect size in the EPM, VCD-treated mice spent less fraction of time in the open arms during the first minute of the test. It should be noted that the rate of acclimatization, a parameter rarely examined in these behavioural tests, as indicated by the minute-by-minute evolution of the measured parameters was highly similar between the two groups.

Our *ex vivo* electrophysiological recordings during the post-menopausal period focused on the dentate gyrus granule cells as these neurons express a high density of δ subunit-containing GABA_A_ receptors, shown by our previous studies^105,106^ to be altered in female mice during changes in ovarian hormone levels. In contrast to the ovarian cycle and pregnancy, we found little differences in the tonic conductances mediated by δ subunit-containing GABA_A_ receptors. Perhaps the much more protracted period of the hormonal imbalance during the post-menopausal period (∼30 weeks) compared with pregnancy (3 weeks) or the ovarian cycle (<1 week) allowed for compensatory changes to take place during this long time. The faster rate of rise of the sIPSCs in VCD-treated mice will have to be further investigated in the regions where the θ and γ oscillations were recorded, as it may be indicative of certain synaptic changes in the interneurons responsible for generating the fast sIPSCs. The typical hormonal changes in the AOF mice included the expected increase in FSH, even though our measurements were done early during the pre-menopausal period (Supplementary Fig. 1). Also, as expected, the post-menopausal bone mass loss was present in our cohort of VCD-treated animals.

In summary, we have identified some profound changes in brain oscillations and their coupling during the pre-menopausal period in the AOF model of human menopause in mice. Equivalent studies are not available for women at a similar hormonal stage, and therefore, our studies open the way to finding new insights into the potential mechanisms underlying the numerous neurological, cognitive and psychiatric alterations surrounding this critical time in women’s lives. We hope that our studies will be followed up in women of menopausal age to yield more insights into the fundamental mechanisms underlying the changes described here, and the extended findings will provide much needed understanding of this highly vulnerable transitional period for women’s health.

## Supplementary Material

fcac166_Supplementary_Data

## Abbreviations

AOF =: accelerated ovarian failure
D =: dark phase of the diurnal cycle (12 h)
EPM =: elevated plus maze
FAC =: frequency–amplitude coupling
FFT =: fast Fourier transform
FSH =: follicle-stimulating hormone
KS =: Kolmogorov–Smirnov test for cumulative distributions
KW =: Kruskal–Wallis test by ranks, or Kruskal–Wallis H test
L =: light phase of the diurnal cycle (12 h)
LFP =: local field potential
mPFC =: medial prefrontal cortex
MWW =: Mann–Whitney–Wilcoxon test, or Mann–Whitney U test, or Wilcoxon rank-sum test, or Wilcoxon–Mann–Whitney test
NREM =: non-rapid eye movement
OF =: open field
PAC =: phase–amplitude coupling
PC =: pyramidal cells
PV + INs =: parvalbumin-positive interneurons
REM =: rapid eye movement
RMS =: root mean squared
SAL =: saline
SPW-R =: sharp-wave ripple
vCA1 =: ventral hippocampal CA1 region
VCD =: 4-vinylcyclohexene-diepoxide
