# Supplementary material for: Altered brain rhythms and behaviour in the accelerated ovarian failure mouse model of human menopause
Source: Brain Commun. 2022 Jun 22;4(4):fcac166. doi: 10.1093/braincomms/fcac166 (PMC9253886; doi:10.1093/braincomms/fcac166)
Supplement: fcac166_Supplementary_Data [file fcac166_supplementary_data.pdf]

Supplementary Material

Altered brain rhythms and behavior in the accelerated ovarian failure mouse model of human menopause

Sophia Vrontou, Alexis Bédécarrats, Xiaofei Wei, Morikeoluwa Ayodeji,
Attila Brassai, László Molnár and Istvan Mody

Materials and Methods

Plasma collection

Mice were anaesthetized with isoflurane and placed on the stereotaxic apparatus without the ear bars. Retro-orbital blood draw was done by penetrating the retro-orbital sinus of both eyes of the mouse with a sterile Pasteur pipette. After the blood collection eye ointment was applied on the eyes. Blood collections were performed 2 days before the start of VCD or saline injections and again 12 weeks later during electrode implantation surgery (Supplementary Figure 1). Blood samples were collected in EGTA coated collection tubes and centrifuged for 10 minutes at 2,000 g. Plasma was stored at -80°C until use. Protein concentration in plasma samples was determined with DC™ Protein Assay (Bio-Rad).

Slice preparation for electrophysiology

Mice were 82-85 weeks old (i.e., 44-47 weeks after the last VCD injections) when the ex vivo experiments were undertaken. They were anesthetized with isoflurane and decapitated following UCLA Chancellor's Animal Research Committee protocol. Horizontal 350 µm thick slices were cut on a Leica VT1200S vibratome in ice-cold N-Methyl-D-Glutamine (NMDG)-based HEPES-buffered solution, containing (in mM): 135 NMDG, 10 D-glucose, 4 $MgCl_2$, 0.5 $CaCl_2$, 1 KCl, 1.2 KH_2PO_4, 20 HEPES, 27 sucrose (bubbled with 100% O_2, pH 7.4, 290-300 mOsm/L). Then, slices were incubated at 32°C in a reduced sodium artificial CSF (ACSF), containing (in mM): NaCl 85, D-glucose 25, sucrose 55, KCl 2.5, NaH_2PO_4 1.25, $CaCl_2$ 0.5, $MgCl_2$ 4, $NaHCO_3$ 26, pH 7.3-7.4 when bubbled with 95% O_2, 5% CO_2. After 30 min low sodium ACSF was substituted for normal ACSF at room temperature, containing (in mM): NaCl 126, D-glucose 10, $MgCl_2$ 2, $CaCl_2$ 2, KCl 2.5, NaH_2PO_4 1.25, Na Pyruvate 1.5, L-Glutamine 1, NaHCO3 26, pH 7.3-7.4 when bubbled with 95% O_2, 5% CO_2. For recording, brain slices were transferred to a submerged recording chamber at 34°C and perfused at 5 ml/min with ACSF. All salts were purchased from Sigma-Aldrich.

Patch clamp recordings and analyses

Slices were visualized under IR-DIC upright microscope (Olympus BX-51WI, 20x XLUMPlan FL N objective) and whole-cell recordings were obtained from either dentate gyrus granule cells or somatosensory pyramidal cell with borosilicate patch pipettes (4-6 MΩ, King Precision Glass) containing of internal solutions (ICS) (in mM): 140 Cs-met, 2 $MgCl_2$ 10 HEPES, 0.2 EGTA, 2 Na_2-ATP, 0.2 Na_2-GTP. The pH of the ICS was adjusted to 7.2 with CsOH and its osmolarity was 285-290 mOsm. ICS were stored at -80°C in 1 ml aliquots. Before each experiment, ICS aliquots were thawed to room temperature and kept on ice during recording. Recordings were obtained using an Axon-patch 200B amplifier (Molecular Devices, San Jose, CA, USA), low-pass filtered at 5 kHz (Bessel, 8-pole) and digitized at 10 kHz with a National Instruments data acquisition board (BNC 2110, National Instruments, Austin, TX, USA). All data were acquired with EVAN (custom-designed LabView-based software). Spontaneous inhibitory post synaptic currents (sIPSCs) were recorded at a holding potential (Vh) of 0 mV. Whole-cell capacitance were estimated from fast transients evoked by a 5 mV voltage command step using lag values of 7 μs and then compensated to 70-80%. The series resistance was monitored before and after the recording, recordings with series resistances >20 MΩ or a change >20% during the recording were excluded.

Tonic and phasic current measurements

A custom written procedure in IGOR Pro 8.0 (Wavemetrics) was used to perform the analysis. An all-points histogram of a randomly selected recording segment of 30 s during the period of interest was plotted. A Gaussian was fitted to the part of the distribution from the minimum value at the left to the rightmost (largest) value of the histogram distribution. The mean of the fitted Gaussian was taken as the tonic current (I_{tonic}). The skewed distribution toward synaptic events (I_{phasic}). This process was repeated for all segments of interest.

IPSCs event detection and measurement

The 30 s recording segment same as the tonic current measurement were detected. Event frequency, averaged amplitude, 10-90% rise times and 63% decay times of detected IPSCs were measure. Decay time was determined by fitting an exponential function to the average sIPSCs decay phase. Detection and analysis were performed using EVAN.

Elevated plus maze (EPM) test

Mice were 56-67 weeks old (i.e., 30-47 weeks after the last VCD injection, Supplementary Figure 1) when they were subjected to the EPM test. Mice were placed in the center of an elevated maze consisting of 2 open arms and 2 closed arms of 50 x 10 cm dimensions (closed arm walls of 40 cm) with the two open and closed arms facing each other arms are opposite to each other. The maze was elevated 50 cm above the floor. The room was evenly red light illuminated, the testing period was 10 min and was performed on the night cycle of the mice. The apparatus was cleaned with water and 70% ethanol between each mouse being tested. Mouse position was captured using a USB infrared camera and iSpy software. The recorded video data were analyzed with custom-

made Igor 6.2 procedures. In brief, the open and closed arms of the maze were divided virtually in centered and peripheral portions and the mouse position was captured every minute (Supplementary Figure 6B) until the end of the 10 minutes sessions.

Open field (OF) test

Mice were 56-67 weeks old (i.e., 30-47 weeks after the last VCD injection, Supplementary Figure 1) when they were subjected to the OF test and placed in the center of an (50 cm x 50 cm) test box. The box was in a room evenly illuminated by red light. The bottom of the box was covered with bedding. The testing period was 10 min and was performed on the night cycle of the mice. The box was cleaned with water and 70% ethanol and fresh bedding was replaced between each mouse being tested. Mouse position was captured using a USB infrared camera and iSpy software. The recorded video data were analyzed with custom-made Igor 6.2 procedures. In brief, the bottom of the box was virtually divided into a center square and a peripheral area of equal areas and the mouse position was captured by the software. The data (distance travelled, speed, mouse position: center vs periphery) were calculated for every minute until the end of the 10 minutes sessions.

CT scans

Five carcasses from the SAL group and 11 carcasses the VCD group were imaged by 3D computerized tomography (CT). Acquisitions were performed with an isometric resolution of 125 µm with the CrumpCAT scanner at the Preclinical Imaging Technology Center of the Crump Institute for Molecular Imaging at UCLA. Bone densities were measured from reconstructed CT scans with the free software AMIDE (developed by UCLA and Stanford University). Illustrations of 3D CT scan reconstructions were made with Avizo software (Thermo Fisher Scientific).

Western blots for FSH measurements

To allow for pre- and post- VCD injection comparison of Follicle Stimulating Hormone (FSH), samples collected before and after saline or VCD treatment were analyzed conjointly with western blots. The use of an ad-hoc western blot method to detect blood circulating proteins[1] is justified in our case by the failure to reliably detect FSH with commercially available ELISA tests (Biomatik kit, Cayman tech kit). A total of seven blots were used to measure FSH (38 samples total from n = 19 animals). The total amount of protein was assessed for each individual sample with a commercial assay (DC™ Protein Assay from Bio-Rad). For each sample 1 µl of plasma was diluted in 14.5 µl phosphate-buffered saline and 14.5 µl of 2X Laemmli sample buffer (Bio-Rad) containing 5% 2-mercaptoethanol (Merck-Sigma-Aldrich). Diluted samples were boiled for 10 min and separated by gel electrophoresis (12% precast polyacrylamide gel, Bio-Rad). Proteins were transferred to a 0.2 µm PVDF membrane (Bio-Rad). Ponceau staining was used to confirm the equal loading of plasma in four out of seven blots. Membranes were blotted overnight in 5% BSA in Tris Buffered Saline with Tween (TBST) containing mouse anti-rabbit polyclonal antibody to FSH used at 1.5 µg/ml, (Cloud-Clone Corp; Cat # PAA830Mu01). Membranes were then incubated for one hour in 5% milk in TBST containing anti-rabbit horseradish peroxidase-labeled antibody, 1:2,000 (GE Healthcare Lifesciences, NA934). Membranes were revealed with Clarity

Western ECL Substrate (Bio-Rad). Chemiluminescence was detected with PXi Multi-Application Gel Imaging System (Syngene). Optical densities were measured with ImageJ (NIH).

Results

Tonic and phasic inhibitions in the dentate gyrus and somatosensory cortex

To explore potential changes in GABAergic inhibitory mechanism after AOF menopause in mice, we performed whole-cell voltage patch clamp recordings using those SAL and VCD treated mice after their in vivo recording and behavioral tests were completed. Spontaneous inhibitory post synaptic currents (IPSCs) were recorded individually from granule cells in the dentate gyrus and pyramidal cells in L2-3 somatosensory cortex.

The recorded IPSCs from dentate gyrus granule cells (DG GC) (Supplementary Figure 5E) and somatosensory cortex pyramidal cells (PC) (Supplementary Figure 5F) were sensitive to 40 μM gabazine (GBZ) in both SAL and VCD treated mice. Tonic and phasic currents were detected and calculated from 30 s epochs under control conditions and in the presence of GBZ. Following subtraction of the values in the presence of GBZ, we obtained the values of tonic current (I_{tonic}) and of the phasic current (I_{phasic}) for each recorded cell as previously published[2]. Supplementary Table 1 lists all tonic current values normalized by whole-cell capacitance of the recorded cell. There is no significant difference between the tonic inhibition of SAL and VCD treatment in both dentate gyrus and somatosensory cortex. Furthermore, we compared the levels of tonic inhibition to the levels of phasic inhibition in the DG and cortex by plotting the values obtained for the two types of inhibition during the same epochs against each other (Supplementary Figure 5C&D). In DG GC recordings, the fitted slope value of phasic vs tonic current in saline group is 0.41 ± 0.09 and is 0.42 ± 0.06 in VCD group, there is no significant difference ($p = 0.4$, MWW test, Cohen's $d = 0.03$) (Supplementary Figure 5C). In the somatosensory cortical PC recordings, the fitted slope of phasic vs tonic current in the SAL group is 0.37 ± 0.17 and is 0.23 ± 0.04 in the VCD group, there is a trend of decreased phasic inhibition at certain tonic inhibition levels in VCD group compared with the SAL group, but without a statistical significance ($p = 0.24$, MWW test, Cohen's $d = 0.43$) (Supplementary Figure 5D).

To further characterize the potential effects of VCD treatment on phasic inhibition, we also used the customary method to detect and analyze IPSCs in our recordings. In Supplementary Table 1 we list all the values of the frequency (Hz), amplitude (pA) and 63 % decay time (T63) of the detected IPSCs from DG GC and cortical PC recordings in the SAL and VCD treated groups. In both DG and somatosensory cortex, the parameters of the detected IPSCs showed no significant differences between SAL and VCD treated groups (Supplementary Table 1), except for the rising time (RT) of detected IPSCs. In Supplementary Figure 5E we show examples of the detected IPSCs and their averaged traces from both SAL and VCD treated groups. The two averaged traces are shown overlapping with the same amplitude and show the way to measure and calculate the 10-90% rising rate (RR) of the detected IPSCs in dentate gyrus: 10-90 % RR = 10-90 % amplitude/10-

90 % rise time (RT). Supplementary Figure 5F illustrates the detected IPSCs and the 10-90 % RR measurement in the cortical PCs. In the DG, the rising phase of the detected IPSCs of GC in VCD group is significantly faster, nearly double of that recorded in the SAL group (Supplementary Table 1, Supplementary Figure 5G). In L2/3 cortical PC, there is no obvious change in the RR of the detected IPSCs between the two groups (Supplementary Table 1, Supplementary Figure 5H).

Behavioral, bone mass, and hormonal alterations in AOF menopausal mice

To further characterize the AOF mouse model of human menopause, we performed a series of behavioral, bone mass and hormonal assays linked to reward-based motivation, anxiety, and other functional changes in human menopause.

We next used the well-established standard of elevated plus-maze (EPM; Supplementary Figure 6A) for assessing anxiety like behaviors in mice[3, 4]. Our software also allowed us to monitor the behavior on the EPM broken down for every 60 s (Supplementary Figure 6B). During the 10 min behavioral task, the VCD and SAL injected mice did not show any difference in the fraction of time spent on the periphery of the open arm or of the closed arm of the maze (Supplementary Figure 6C&D; Cohen's d = 0.45 for C and d=0.36 for D). After binning the behavior in the maze in 60 s intervals, it was revealed that during the first 60 s of the assay VCD mice tend to spend less time than the SAL mice on the periphery of the open arm of the maze ($p>0.05$, MWW test, Cohen's d = 0.96) and more time in the closed arm ($p>0.05$, MWW test, Cohen's d = 0.99). Although not statistically significant, these large effect sizes may indicate a different behavior between the SAL and VCD mice. During the EPM assay there is a continuous conflict between the innate avoidance of mice for open areas and their natural tendency to explore novel environments (Supplementary Figure 6E&F). The latter drive might overcome the first as time proceeds and therefore might explain why only during the first 60 s of the test a contrast between VCD and SAL mice is evident.

We also performed the open field (OF) exploration assay (Supplementary Figure 7A&B) that evaluates, general locomotor activity novel environment exploration and anxiety-related behavior. The VCD mice traveled a somewhat longer distance (Supplementary Figure 7C, $p>0.05$, MWW test, Cohen's d = 0.5) with a corresponding higher speed (Supplementary Figure 7D, $p>0.05$, MWW test, Cohen's d = 0.76) than the SAL mice during the entire 10 min in the OF test. In addition, the VCD mice spent less time in the center of the arena, since they showed a smaller center to periphery ratio that albeit statistically not significant, it showed a large effect size (Supplementary Figure 7E, $p>0.05$, MWW test, Cohen's d = 1.02). The SAL and VCD animals showed a similar rate of decline of the distance traveled and speed over the 10 min of the test (Supplementary Figure 7F&G), and a similar rate of increase in the time spent in the center box (Supplementary Figure 7H).

Osteoporosis, a bone disease characterized by a loss of bone mass is a consequence of menopause in women[5], and in VCD treated mice[6]. Furthermore, bone loss appears to correlate with cognitive decline in menopausal women[7]. Therefore, we wanted to know whether AOF induced this well reported consequence of the menopausal state in our VCD treated animals. Therefore, we measured the bone density on CT-scans of the carcasses of the animals used in our study.

Supplementary Figure 8 shows a typical loss of bone mass in the lumbar 4 (L4) vertebra of a VCD treated mouse compared to the L4 bone density of a SAL injected mouse. Similar decreases in bone density could also be measured in L6 and T12 vertebrae (Supplementary Table 2).

In contrast to ovariectomy, a distinguishing feature of the AOF model of menopause is the hormonal changes accurately mirroring the human condition[8]. One of these characteristic alterations is the increase in the follicle stimulating hormone (FSH), that has been shown also to have wide metabolic effects[9]. To detect changes in FSH levels associated with the induction of a menopausal state we ran plasma samples collected before and 12 weeks after the SAL or VCD treatment side by side into electrophoresis gels. Subsequently we detected the relative changes in FSH with western blots (Supplementary Figure 9A). Consistent with AOF, FSH levels in plasma samples from mice injected with VCD (n = 11 pairs) were statistically higher than pre-injection levels (VCD pre vs VCD post, $p = 0.0029$) (Supplementary Figure 9B). Conversely, FSH levels detected from plasma samples from mice collected before and after saline injection (n = 8 pairs) were not significantly different between the two groups (SAL pre vs SAL post; $p = 0.6406$, Supplementary Figure 9B). Changes in total amount of protein in plasma were not significantly when we compared the effect of the saline injection ($p = 0.8438$), or the VCD injection ($p = 0.4102$, Supplementary Figure C). Supplementary Figure 10 shows the full extent of the western blots used for the measurements depicted in Supplementary Figure 9.

References

1. Horowitz AM, Fan X, Bieri G, et al. Blood factors transfer beneficial effects of exercise on neurogenesis and cognition to the aged brain. *Science*.2020;369:167-173.
2. Glykys J, Mody I. The main source of ambient GABA responsible for tonic inhibition in the mouse hippocampus. *JPhysiol*.2007;582:1163-1178.
3. Haller J, Alicki M. Current animal models of anxiety, anxiety disorders, and anxiolytic drugs. *Current opinion in psychiatry*.2012;25:59-64.
4. Lezak KR, Missig G, Carlezon WA, Jr. Behavioral methods to study anxiety in rodents. *Dialogues in clinical neuroscience*.2017;19:181-191.
5. Karlamangla AS, Burnett-Bowie SM, Crandall CJ. Bone Health During the Menopause Transition and Beyond. *Obstetrics and gynecology clinics of North America*.2018;45:695-708.
6. Wright LE, Christian PJ, Rivera Z, et al. Comparison of skeletal effects of ovariectomy versus chemically induced ovarian failure in mice. *J Bone Miner Res*.2008;23:1296-303.
7. Bliuc D, Tran T, Adachi JD, et al. Cognitive decline is associated with an accelerated rate of bone loss and increased fracture risk in women: a prospective study from the Canadian Multicentre Osteoporosis Study. *J Bone Miner Res*.2021;36:2106-2115.
8. Marongiu R. Accelerated Ovarian Failure as a Unique Model to Study Peri-Menopause Influence on Alzheimer's Disease. *Frontiers in aging neuroscience*.2019;11:242.
9. Taneja C, Gera S, Kim SM, Iqbal J, Yuen T, Zaidi M. FSH-metabolic circuitry and menopause. *Journal of molecular endocrinology*.2019;63:R73-R80.
10. Buzsáki G. Hippocampal sharp wave-ripple: A cognitive biomarker for episodic memory and planning. *Hippocampus*.2015;25:1073-188.

11. Safaryan K, Mehta MR. Enhanced hippocampal theta rhythmicity and emergence of eta oscillation in virtual reality. *Nat Neurosci*.2021;24:1065-1070.

Tables

Supplementary Table 1. Summary of tonic GABAergic inhibition and properties of detected sIPSCs in dentate gyrus granule cells (DG GC) and somatosensory cortical layer 2-3 pyramidal cells (Cortex L2/3 PC) recorded in slices prepared from SAL and VCD injected mice. The p-values for the MWW test between the mean SAL and VCD values are indicated. The effect size refers to the Cohen's d.

	Frequency (Hz)			
Mean	9.92	7.56	20.52	18.49
SD	5.44	5.14	13.06	11.39
WMW p	0.18		0.95	
Cohen's d	0.45		0.17	
	Amplitude (pA)			
Mean	20.07	22.47	31.83	27.18
SD	2.00	4.46	12.52	5.22
WMW p	0.09		0.68	
Cohen's d	0.69		0.48	
	63% Decay Time (T63) (ms)			
Mean	7.61	7.25	6.21	5.58
SD	1.84	2.10	3.50	1.75
WMW p	0.59		>0.99	
Cohen's d	0.18		0.23	
	10-90% Rate of Rise (pA/ms)			
Mean	11.99	25.31	48.82	37.98
SD	6.37	19.26	42.23	18.86
WMW p	0.03		>0.99	
Cohen's d	0.93		0.33	

Supplementary Table 2. Bone density values for three types of vertebrae measured by CT scans in SAL and VCD injected mice. The p-values for the MWW test between the mean SAL and VCD values are indicated. The Cohen's d values (effect size) are also indicated.

Vertebra	Animal	Mean	SEM	n	p	Cohen's d
L4	SAL	955.4	15.4	5	0.0018	1.92
	VCD	892.7	9.3	11		
L6	SAL	885.5	6.7	5	0.0005	2.42
	VCD	834.3	7.8	11		
T12	SAL	998.0	10.5	5	0.0275	1.45
	VCD	933.9	17.5	11		

Supplementary Figures and Figure Legends

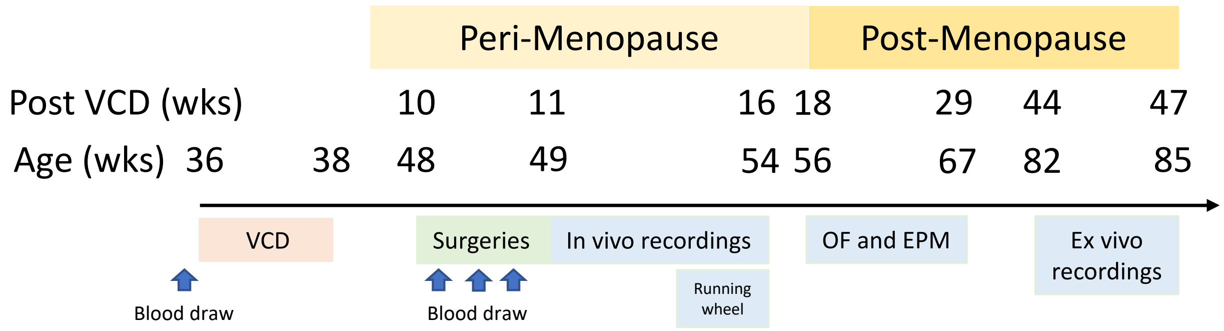

Supplementary Figure 1. The timeline of the VCD-induced AOF and our experimental protocols. The age of the animals, the timing of the VCD injections and of the experimental manipulations are clearly indicated. The peri- and post-menopause periods are adapted from reference 49 (main text). It is clear that our in vivo LFP recording sessions and voluntary wheel running tests all took place during the peri-menopause while the behavioral tests and ex vivo electrophysiology was carried out during the post-menopause.

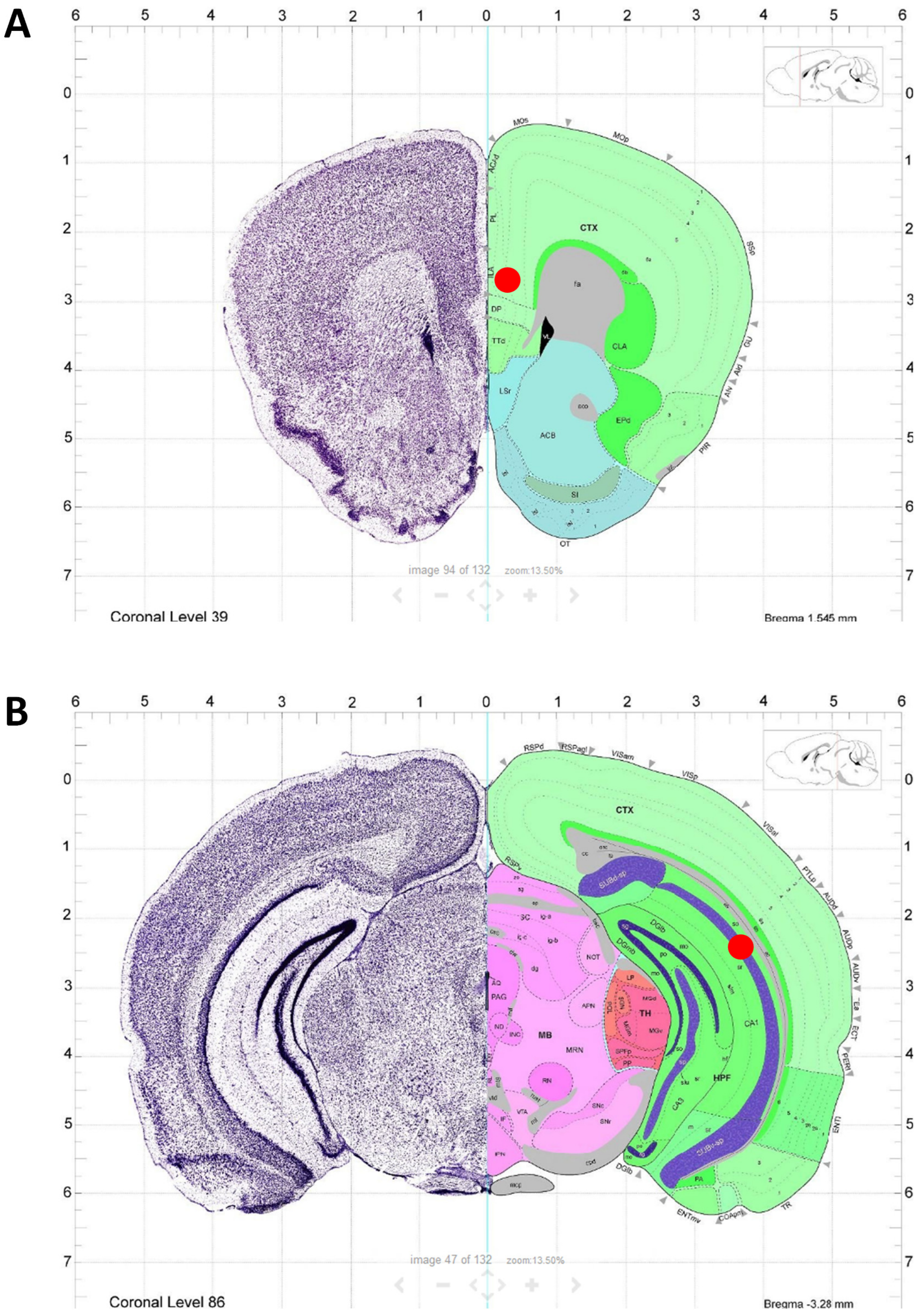

Supplementary Figure 2. Electrode placement in the ventral hippocampal CA1 (vCA1) and the infralimbic part of the mPFC. A. Section from the ALLEN Mouse Brain Atlas, Version 2

(2011), Allen Institute for Brain Science marking the position of the implanted electrode in the mPFC. **B.** Section from the same atlas that marks the position of the implanted vCA1 electrode. Images were taken from: http://mouse.brain-map.org/experiment/siv?id=100142143&imageId=102162175&imageType=atlas&initImage=atlas&showSubImage=y&contrast=0.5,0.5,0,255,4

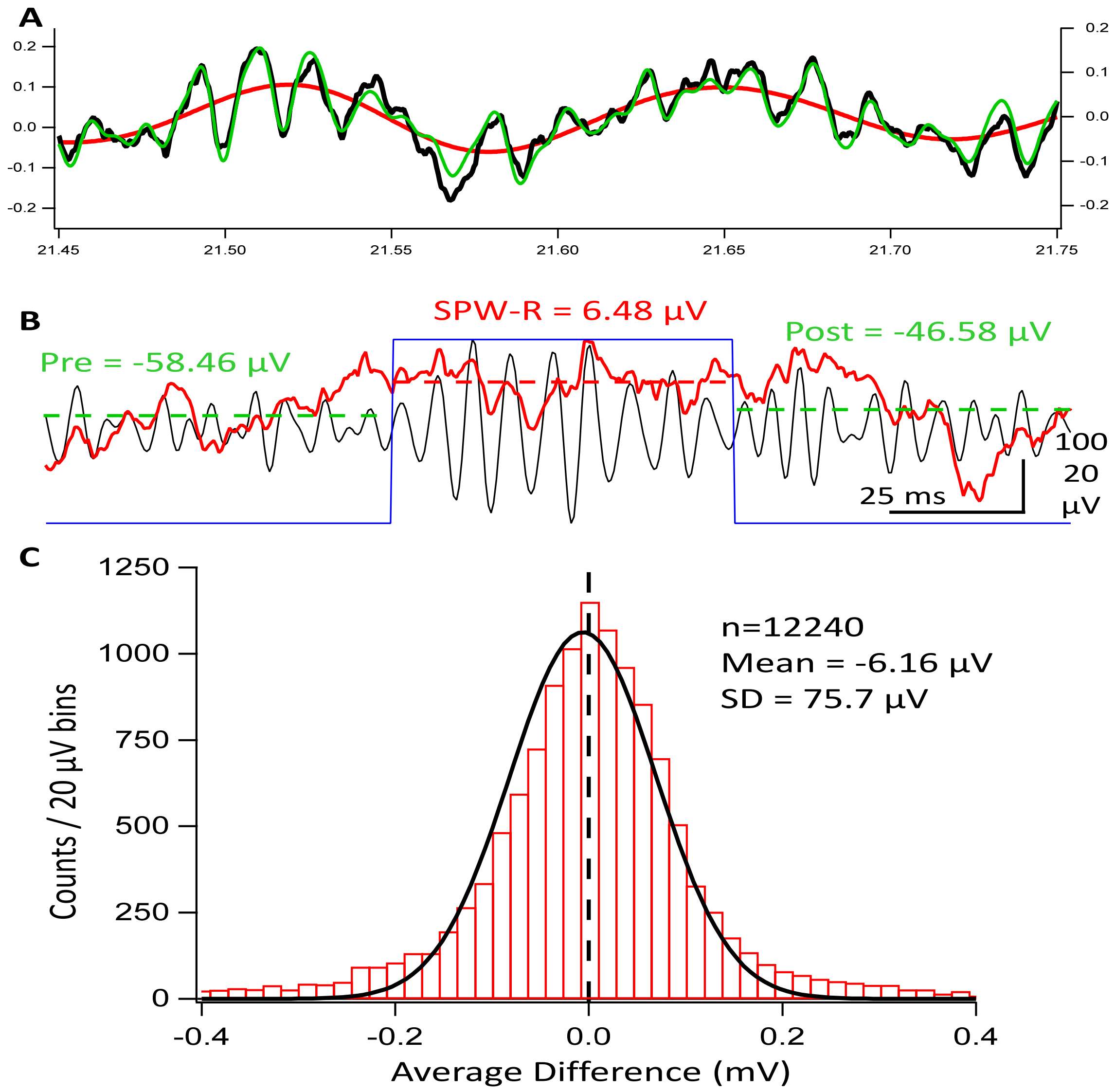

Supplementary Figure 3. Signal filtering and verification of electrode placement in the ventral hippocampal CA1 (vCA1) pyramidal cell layer. **A**. 300 ms long LFP recording (*black*) with superimposed FIR filtered traces in θ (red) and θ+γ (*green*) band-widths showing no phase shifts. **B**. Detection of a 67 ms long SPW-R (*blue*), raw signal (*red*) and 125-250 Hz filtered (*black*) traces. The average values of the similar duration pre- and post- SPW-R epochs are shown. **C**. Histogram of the averaged differences between the SPW-R and its pre- and post- epochs for >12000 events detected during 12 hrs of recording. The mean of the distribution is close to 0, indicating that there is no "wave" structure in the SPW-R, i.e., it has been recorded close to the pyramidal cells layer[10, 11].

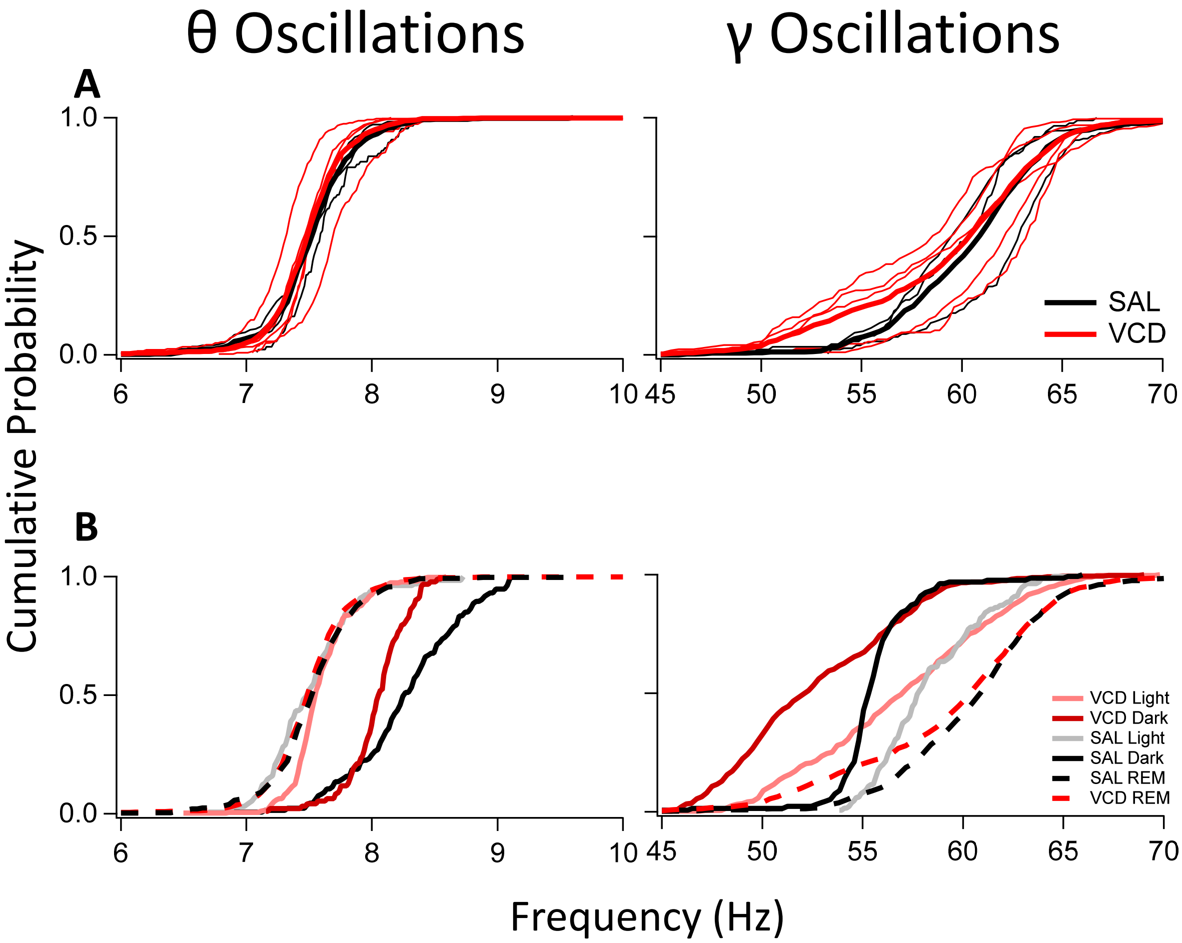

Supplementary Figure 4. θ and γ oscillation frequencies during REM sleep. A. Cumulative probability plots of the hourly average θ oscillation frequencies (*left panel*) and γ oscillation frequencies (*right panel*) recorded during REM sleep over several days from SAL (n=3) and VCD (n=5) animals. The thick lines are the grand average distributions. There are no significant differences between the distributions between the two groups (Kolmogorov-Smirnov, K-S test $p>0.001$). **B.** Comparison of the θ oscillation frequencies (*left panel*) and γ oscillation frequencies (*right panel*) recorded during REM periods with the same oscillations recorded during the light and dark phases of the circadian cycle during AWAKE periods. For both SAL and VCD groups, the cumulative distributions of θ oscillation frequencies during REM sleep are no different than those recorded during AWAKE periods during the light cycle (K-S test $p>0.001$). In contrast, for both SAL and VCD groups the γ oscillation frequency cumulative probability plots are shifted to the right, towards larger frequencies, during REM sleep compared to AWAKE periods, regardless of the diurnal cycle (K-S test $p<0.001$).

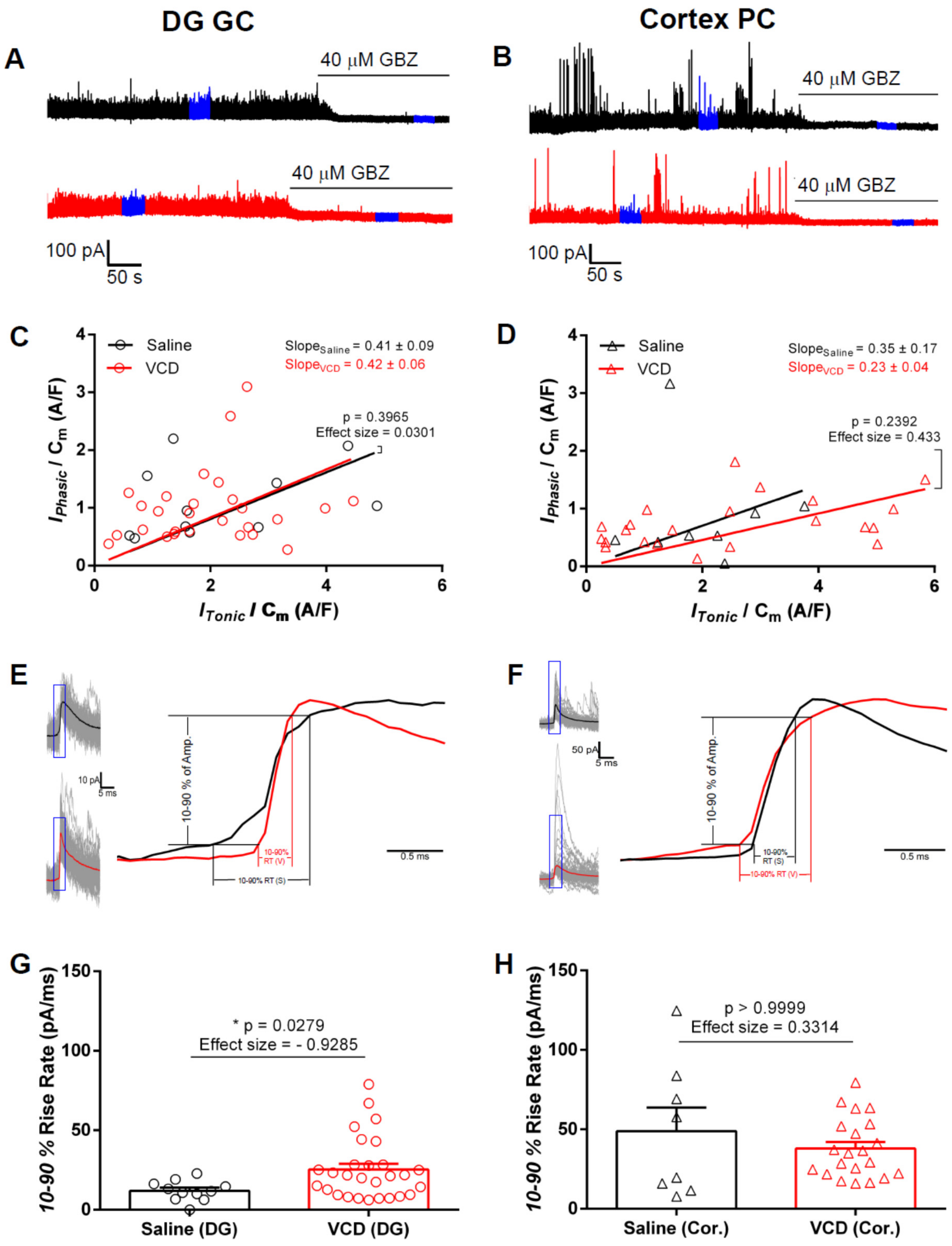

Supplementary Figure 5. Inhibitory post synaptic current (IPSCs) recordings from dentate gyrus (DG) granule cells (GCs) (left column) and somatosensory cortex layer 2/3 pyramidal cells (PCs) (right column). A, B. Raw traces of IPSCs recorded from DG GC (A) and Cortex

PC (B), most of IPSCs can be blocked after applying 40 mM GBZ. Totally 4 Saline treated control mice (traces in black) and 10 VCD treated mice (traces in red) were used. 30 s recording section (blue) in each period were analyzed. **C, D.** Relationship between the phasic and tonic currents. All tonic and phasic currents were normalized by whole-cell capacitance (Cm). There are no significant differences between the slopes of the lines fitted to the SAL and VCD groups in both DG and cortex. The fitted slopes of the lines, the p values and Cohen's d values are indicated. **E, F.** *Left panels*: Example traces of IPSCs of from one DG GC and one cortical L2/3 PC in a SAL (averaged trace in *black*) and a VCD (averaged trace in *red*) animal, respectively; *Right panels*: superimposition of the average traces from the left, and the measurement of the 10-90% rate of rise (RR) by dividing the 10-90% amplitude with its corresponding rise time (10-90% RT). S: saline. V:VCD. **G, H.** Plot of the RR of detected IPSCs. In DG GC recordings, the RR in VCD group (*red*; n=28 cells) is significantly larger than in the SAL group (*black*; n=11 cells; G); In cortical PC recordings, the RR is not significantly different between the two groups (SAL: n=8 cells; VCD: n=21 cells; H). The p values (MWW test) and Cohen's d values are indicated.

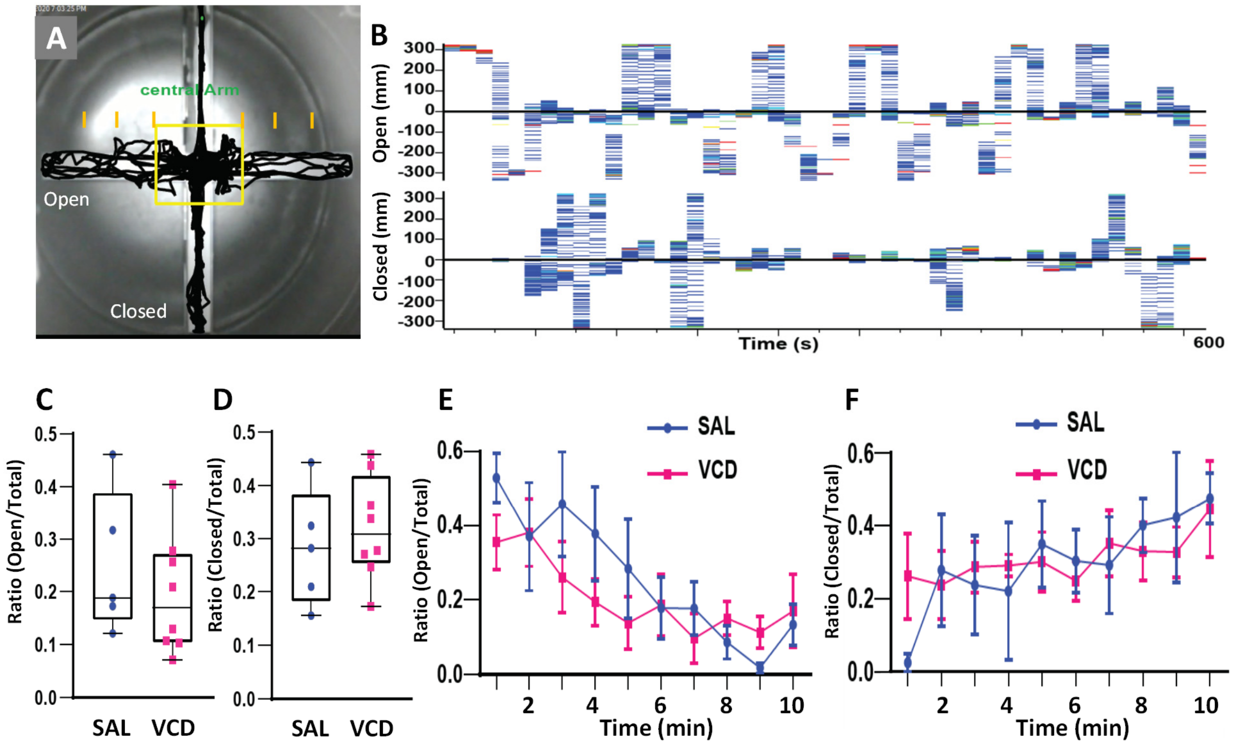

Supplementary Figure 6. Anxiety related behavior of the VCD mice using the elevated plus maze (EPM) test. A. Locomotor trail for one example mouse in the EPM during the entire10 min of the assay. The open arms in the maze are in horizontal direction and each arm is divided in predefined segments that extend 300, 200 and 100 mm (yellow bars) from the center which is designated as zero. **B.** Histogram of the density of the trajectory in each arm segment binned every 10 s, throughout the course of the test, for the representative mouse is shown in A. **C.** VCD mice display a tendency to spend more less fraction of time in the open arms ($p>0.05$, Cohen's d=0.36) and more fraction of time in the closed arms (**D**) ($p>0.05$, Cohen's d=0.45). **E.** This inclination is especially amplified during the first minute of their behavior, as indicated by the high effect sizes for the reduced time fraction in the open arms ($p>0.05$, Cohen's d=0.96) and the increased time fraction spent in the closed arms (**F**) ($p>0.05$, Cohen's d=0.99). Note that as time passes, both SAL and VCD mice spend less fraction of time in the open arms and gradually more fraction of time in the closed arms. Total time was calculated as the time the mouse spent in both open and closed peripheral arms summed with the time spent in the centers of the open and closed arms. Box and whisker plots show the range of individual data points, with the interquartile spread as the box, the median as the line bisecting the box and Tuckey style whiskers. Data were obtained from n=5 SAL and n=8 VCD mice. All statistics were done using the MWW test.

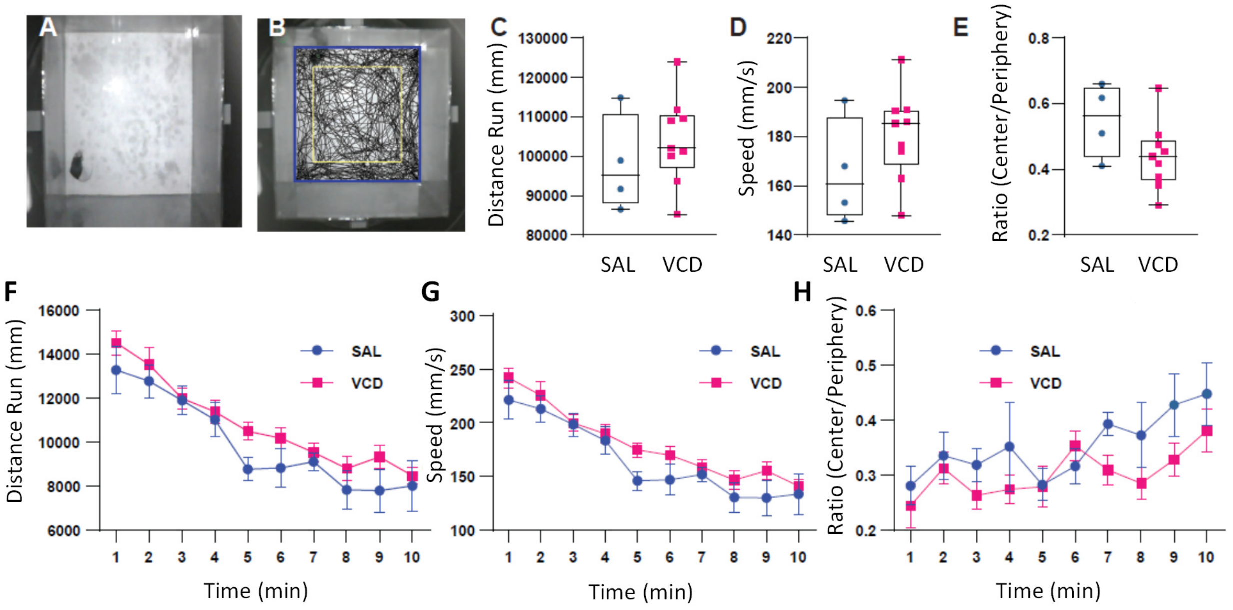

Supplementary Figure 7. VCD treated mice show a tendency for increased anxiety in the open field (OF) test. A. Image of a mouse in the OF arena. **B.** Movement trajectory of the mouse shown after completion of the OF behavior. Blue square indicates the peripheral borders of the arena and the yellow square is the perimeter of the designated center (the areas of center and periphery are equal). Over the 10 min of the test, VCD mice tend to cover a longer distance (**C**), have a higher speed (**D**) than their SAL counterparts, with medium effect sizes but non-significant probabilities (Cohen's d=0.76 and 0.51, respectively, and p>0.05. **E.** VCD mice also tend spend a decreased, albeit not statistically significant time in the central area (p>0.05), the high Cohen's d=1.02 indicates a large effect size. **F-H.** Minute-by-minute breakdown of the average (± SEM) speed, distance run, and center to periphery ratios of the VCD and SAL mice during the 10 min course of the OF assay. Note that in both groups of mice the speed and distance decrease with time, while the center to periphery ratios increase, most likely indicating acclimatization to the OF arena. Box and whisker plots show the range of individual data points, with the interquartile spread as the box, the median as the line bisecting the box and Tuckey style whiskers. All data were generated from n=4 SAL and n=9 VCD mice. MWW test was used for the statistics.

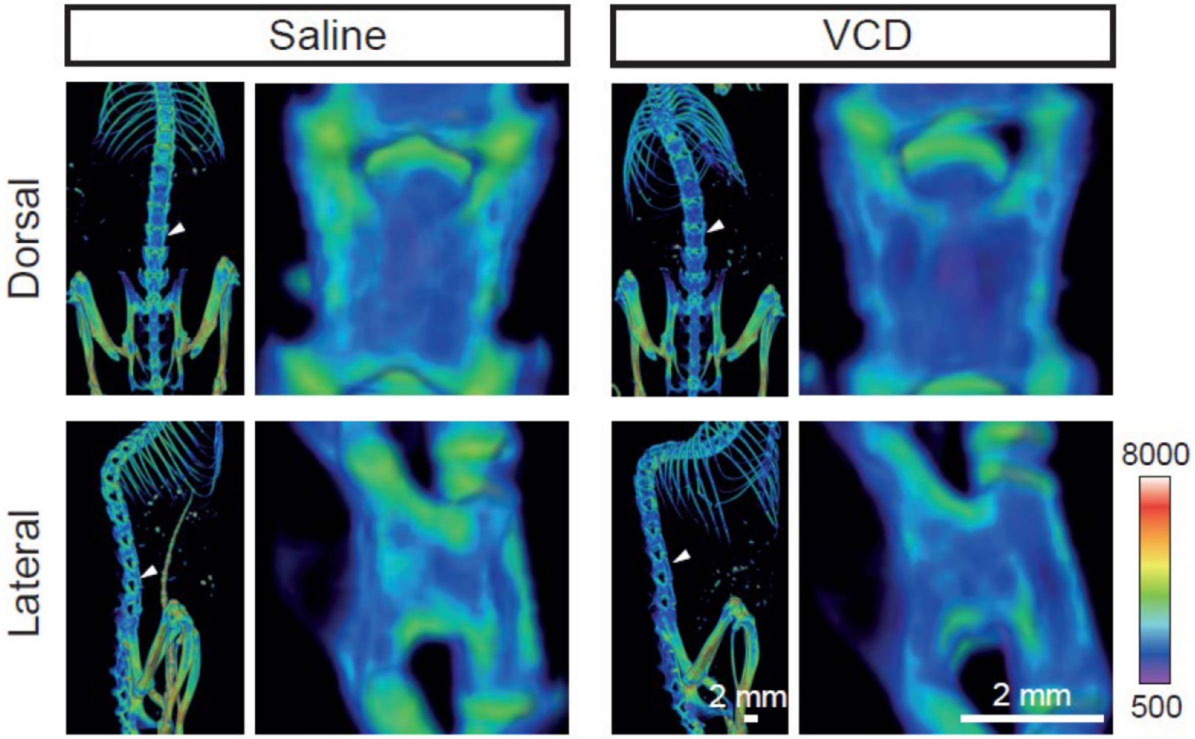

Supplementary Figure 8. Bone mass alterations in AOF menopausal model mice. Reconstructed CT scans from a SAL injected (left) and a VCD injected animal (right). Dorsal and lateral views of the Thoraco-lumbar region and enlarged lumbar vertebrae 4 (L4) are shown side by side in both animals. The white arrow heads on the left panels indicate L4. A linear rainbow color scale is used to visualize change in bone density (Hounsfield units).

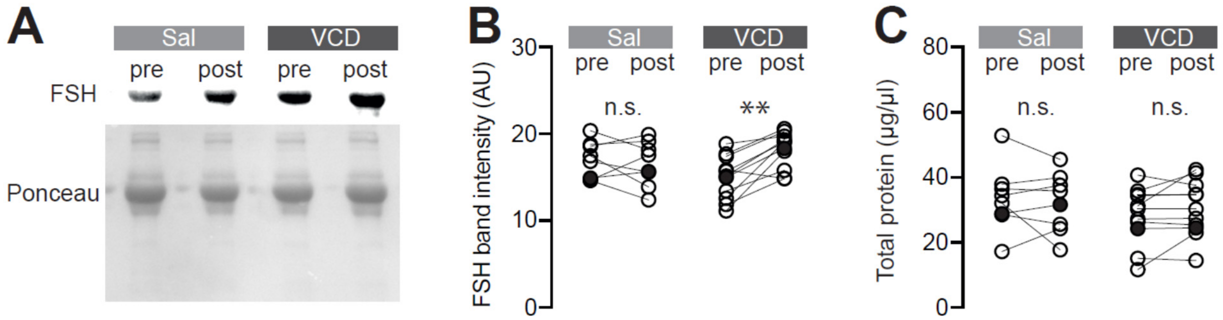

Supplementary Figure 9. VCD injections increase FSH levels in blood plasma in adult female mice. A. Representative western blot and matching Ponceau red staining before and three months after VCD or saline treatment. The same volume of plasma was loaded in consecutive lanes. **B.** Paired measurements of FSH band intensity for the two groups. **C.** Paired measurements of total protein contained in plasma samples. Black plain circles represent values from the example of FSH bands illustrated in (A). $^{**}p < 0.01$, n.s., non-significant, paired Wilcoxon signed rank test.

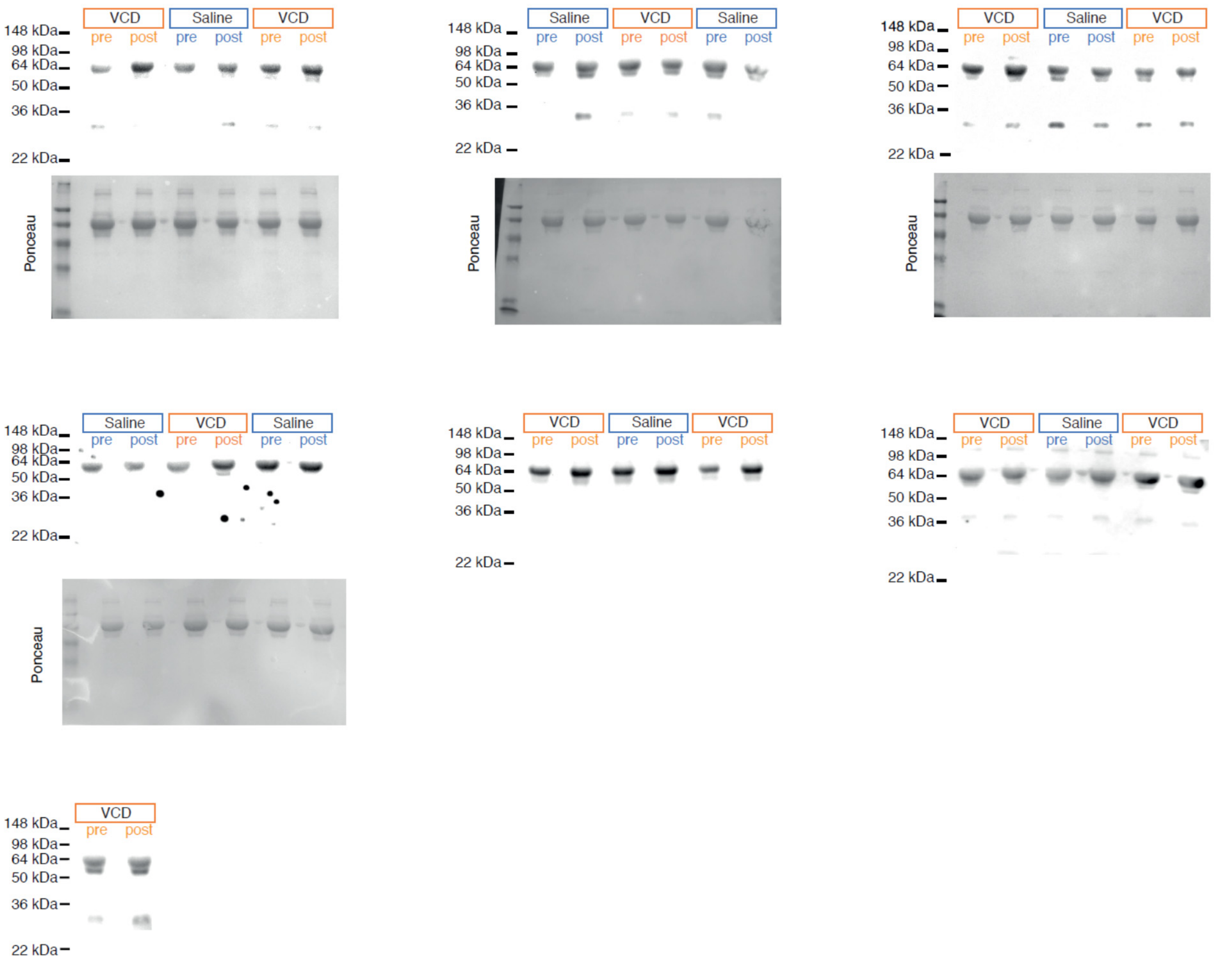

Supplementary Figure 10. Extended western blots for the data shown in Supplementary Figure 9.
